# Modulation of Ambient Temperature-Dependent Flowering in *Arabidopsis thaliana* by Natural Variation of *FLOWERING LOCUS M*

**DOI:** 10.1371/journal.pgen.1005588

**Published:** 2015-10-22

**Authors:** Ulrich Lutz, David Posé, Matthias Pfeifer, Heidrun Gundlach, Jörg Hagmann, Congmao Wang, Detlef Weigel, Klaus F. X. Mayer, Markus Schmid, Claus Schwechheimer

**Affiliations:** 1 Plant Systems Biology, Technische Universität München, Freising, Germany; 2 Department of Molecular Biology, Max Planck Institute for Developmental Biology, Tübingen, Germany; 3 Plant Genome and Systems Biology, Helmholtz Zentrum München, German Research Center for Environmental Health, Neuherberg, Germany; University of Lausanne, SWITZERLAND

## Abstract

Plants integrate seasonal cues such as temperature and day length to optimally adjust their flowering time to the environment. Compared to the control of flowering before and after winter by the vernalization and day length pathways, mechanisms that delay or promote flowering during a transient cool or warm period, especially during spring, are less well understood. Due to global warming, understanding this ambient temperature pathway has gained increasing importance. In *Arabidopsis thaliana*, *FLOWERING LOCUS M* (*FLM*) is a critical flowering regulator of the ambient temperature pathway. *FLM* is alternatively spliced in a temperature-dependent manner and the two predominant splice variants, *FLM-ß* and *FLM-δ*, can repress and activate flowering in the genetic background of the *A*. *thaliana* reference accession Columbia-0. The relevance of this regulatory mechanism for the environmental adaptation across the entire range of the species is, however, unknown. Here, we identify insertion polymorphisms in the first intron of *FLM* as causative for accelerated flowering in many natural *A*. *thaliana* accessions, especially in cool (15°C) temperatures. We present evidence for a potential adaptive role of this structural variation and link it specifically to changes in the abundance of *FLM-ß*. Our results may allow predicting flowering in response to ambient temperatures in the *Brassicaceae*.

## Author Summary

Plants control their flowering time in response to the temperatures of their environment, e.g. in response to the experience of winter or in response to cold and warm ambient temperatures experienced during spring. The knowledge about the evolutionary adaptation of plants to changing ambient temperatures is at present very limited. Understanding the latter is, however, becoming increasingly important due to the temperature changes associated with global warming and the anticipated changes in flowering time in ecosystems and agricultural systems. Here, we uncover an evolutionarily conserved molecular mechanism employed by *Arabidopsis thaliana* ecotypes for the adaptation of flowering time to cool temperatures. This structural change in the architecture of the gene *FLOWERING LOCUS M* can be found in multiple *A*. *thaliana* natural accessions and the knowledge gained in our study may be used to predict or modify flowering time in plants related to *A*. *thaliana* in the future.

## Introduction

In plants, fertilization and reproduction are directly linked to the seasonal onset of flowering. Plants enter the reproductive phase when environmental conditions are favorable for seed set and thus reproduction. Since day length and temperature as well as temperature changes throughout the year provide the crucial information about the passage of the seasons and the environment, plants sense these cues for the adjustment of their flowering time [1]. Proper flowering time and reproductive success of a given species or ecotype, on the one side, and the differences in flowering time between species or ecotypes, on the other, are the result of the differential integration of temperature and day length information.

The vernalization and the ambient temperature pathways control temperature-dependent flowering in plants. Whereas vernalization requires long periods (weeks) of cold, usually below 10°C, as experienced during the winter [2], the ambient temperature pathway modulates flowering in response to short-term (days) temperature changes in the range between 12°C and 27°C [3–5]. In *A*. *thaliana*, the central mechanism of accelerating flowering in response to prolonged cold is achieved by repression of the negative regulator *FLOWERING LOCUS C* (*FLC*), a MADS-box transcription factor [6–9]. Different mechanisms than in *A*. *thaliana* control vernalization in cereal crops such as wheat and barley, and the activity or inactivity of the vernalization pathway determines the flowering behavior of their winter and spring varieties [10, 11]. To date, the understanding of the vernalization pathway in *A*. *thaliana* is already well advanced and it is possible to make predictions on the vernalization requirement based on the plants’ genotypes [12, 13].

In contrast, the complexities of ambient temperature sensing are just beginning to be understood [5, 14, 15]. The finding that loss-of-function mutations of the gene *FLM* (*FLOWERING LOCUS M*) reduce the temperature-sensitivity of flowering in *A*. *thaliana* accessions suggested that this MADS-box transcription factor acts as a repressor in the ambient temperature pathway [16–18]. The molecular understanding of *FLM* is complicated by the fact that the *FLM* gene is alternatively spliced into at least four splice forms [18]. *FLM-ß* and *FLM-δ*, which result from the alternative use of the two exons 2 (*FLM-ß*) and 3 (*FLM-δ*), represent the two predominant splice variants in the Columbia-0 (Col-0) reference accession [19, 20]. The observation that the abundance of *FLM-ß* declines from 16°C to 27°C while the abundance of *FLM-δ* increases over the same temperature range has motivated experiments to examine the effects of the *FLM-ß* and *FLM-δ* isoforms in isolation in a *flm-3* loss-of-function background. These experiments indicated that the expression of the low temperature-abundant *FLM-ß* and the warm temperature-abundant *FLM-δ* can repress and promote flowering, respectively, and consequently a model was established according to which changes in the relative abundance of *FLM-ß* and *FLM-δ* control flowering time in response to changes in ambient temperature [19].

FLM directly interacts with several other MADS-box transcription factors to control flowering through the expression of flowering time genes such as *FT* (*FLOWERING LOCUS T*) and *SOC1* (*SUPPRESSOR OF OVEREXPRESSION OF CO1*) [19–21]. SVP (SHORT VEGETATIVE PHASE) is an important FLM interaction partner and, in this context, the flowering-repressive activity of FLM-ß and the flowering-promoting activity of *FLM-δ* have been explained by the differential effects of the FLM-SVP interactions [19]: It was proposed that a DNA-binding heterodimer of FLM-ß with SVP represses flowering by repressing *FT* and *SOC1* expression. Conversely, FLM-δ could sequester SVP into an inactive complex that thereby indirectly promotes *FT* and *SOC1* expression and consequently flowering. Although this experimentally validated model is very intriguing, it is at present not known whether the alternative splicing of *FLM* plays a role in flowering time adaptation in natural accessions of *A*. *thaliana*.

There is increasing evidence for global warming due to climate change [8]. Temperature changes by only a few centigrade (°C) can already lead to ecological and physiological constraints that have negative impacts on agricultural production systems [22–24]. Thus, there is a need to better understand the ambient temperature pathway and to integrate this understanding in plant breeding programs [25]. Here, we identify a structural polymorphism in the first intron of *FLM* as being causative for the early flowering time of the *A*. *thaliana* accession Killean-0 (Kil-0). This structural polymorphism is present in several additional accessions and directly affects *FLM* transcript abundance, splicing, and flowering. We further correlate the abundance of the *FLM-ß* and *FLM-δ* splice variants with flowering behavior in several *A*. *thaliana* accessions and reveal an important role of intron 1 for *FLM* gene expression and a predominant role of *FLM* in flowering time control.

## Results

### Killean-0 is an early flowering accession

**Fig 1:**
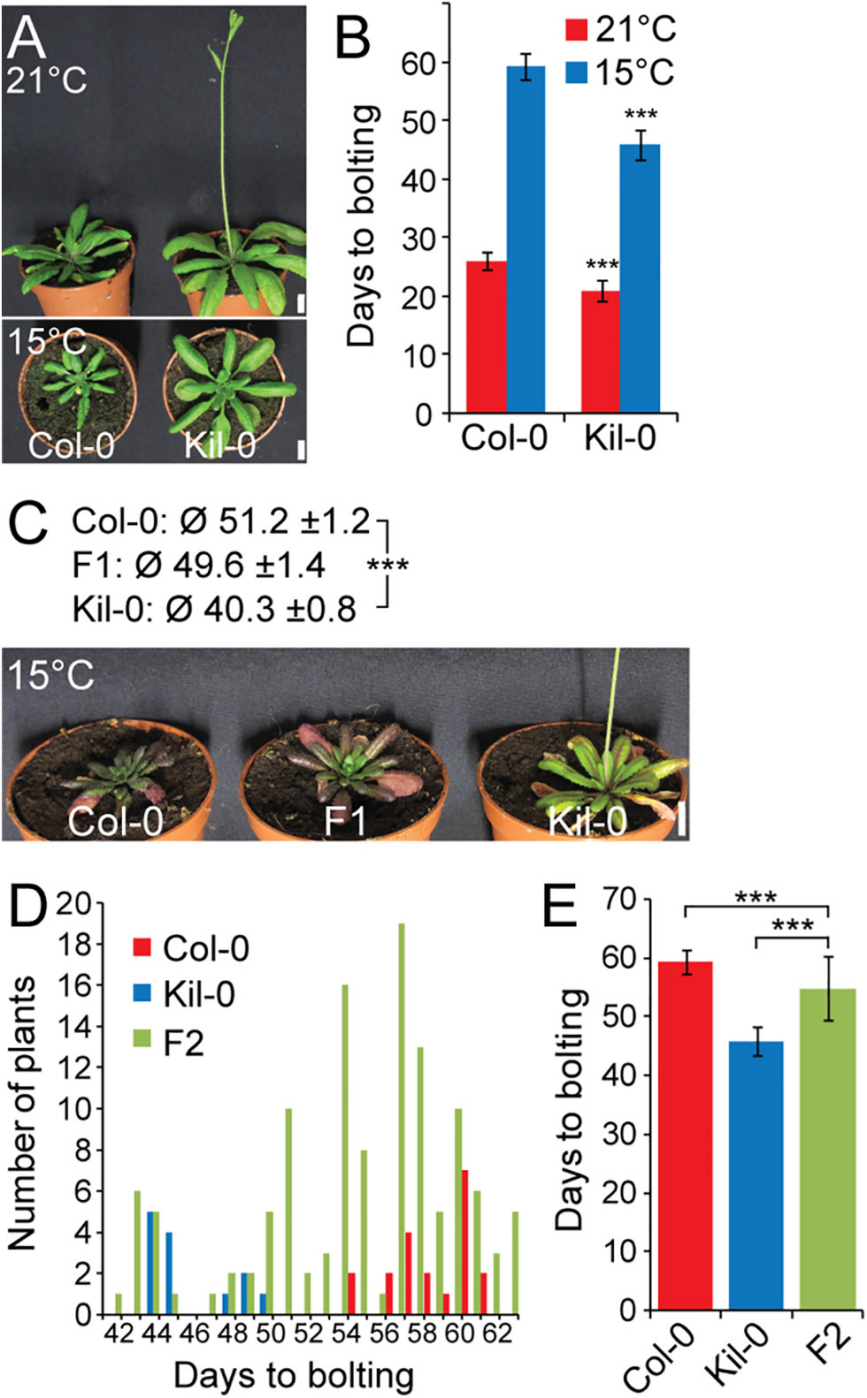
Early flowering is controlled by one major recessive locus in Kil-0. **(A)** Representative photographs and **(B)** quantitative flowering time analysis (days to bolting ± SD) of Col-0 and Kil-0 grown under continuous light at 21°C and 15°C. **(C)** Representative photographs of 50 day-old plants of Col-0, Kil-0, and their F_1_ hybrid grown under continuous light at 15°C. The average days to bolting ± SD are indicated above the photograph. **(D)** Distribution of flowering time and **(E)** average days to bolting ± SD in a population of 124 F_2_ segregants from a Col-0 x Kil-0 hybrid as well as among Col-0 (n = 20) and Kil-0 (n = 15) plants. Student’s t-tests were performed in comparison to the wild type unless indicated otherwise in the figure: *** = p ≤ 0.001. Scale bar = 1 cm.

To understand the variation in flowering time in response to temperature, we compared the flowering behavior of a collection of *A*. *thaliana* accessions at 15°C and at 21°C. In this analysis, our attention was drawn to the Scottish accession Killean-0 (Kil-0), which flowered two weeks earlier than the Columbia-0 (Col-0) reference when grown at 15°C but only one week earlier at 21°C (Figs 1A, 1B, S1A and S1B). The vernalization pathway could potentially contribute to the early flowering behavior of Kil-0 at 15°C but we detected only minor flowering time effects after a six-week vernalization treatment (S1C Fig). These flowering time effects were similar to those observed in the reference Col-0 and confirmed also the results from previous surveys that had classified Kil-0 as a summer annual [12, 26]. Since the expression of the major vernalization-responsive gene *FLC* was also as strongly reduced in Kil-0 as in the summer annual accession Col-0 (S1D Fig), we concluded that the temperature-dependent early flowering phenotype of Kil-0 at 15°C was vernalization-independent.

### *FLM* is the causative locus for early flowering in Kil-0

**Fig 2:**
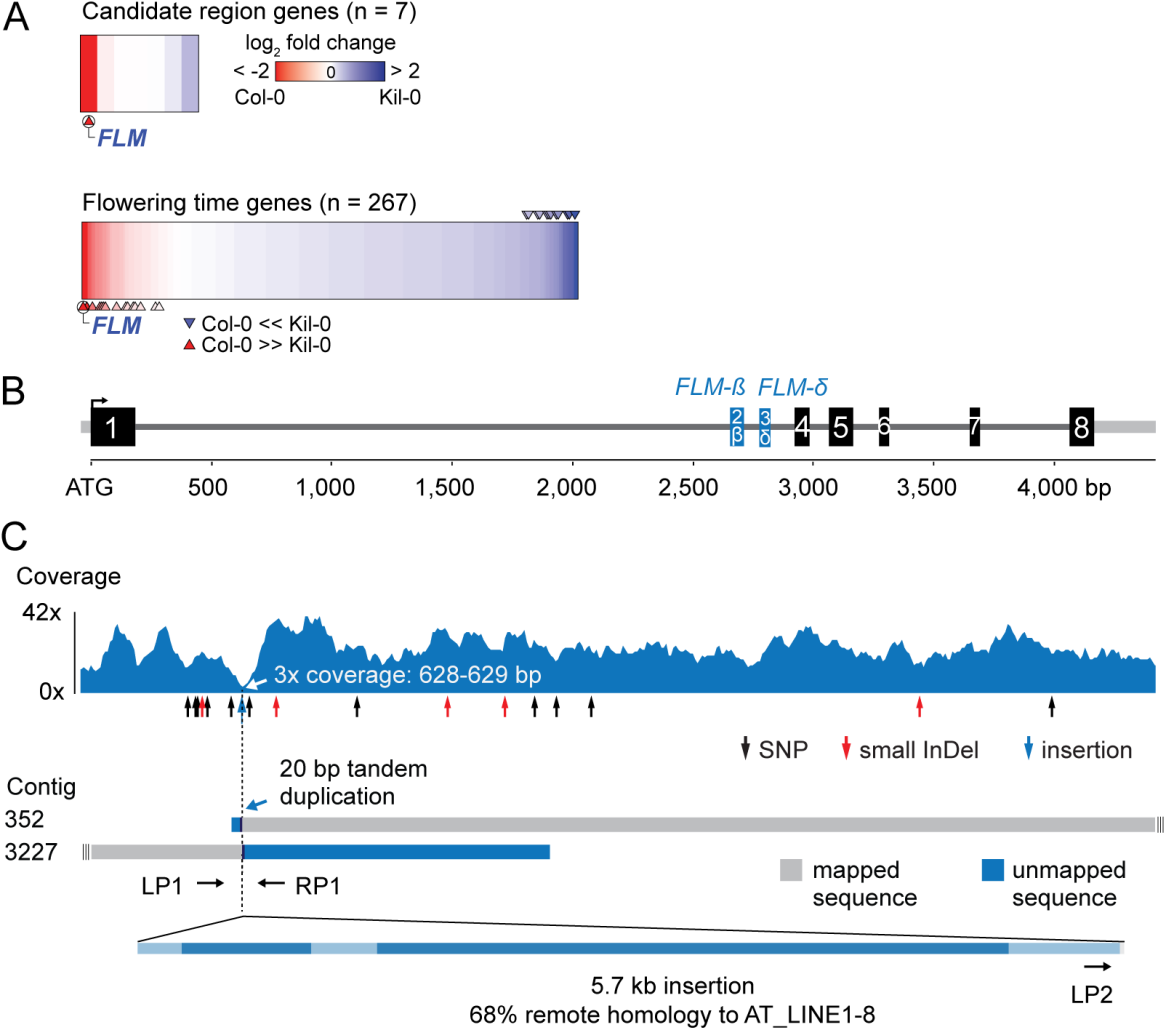
Identification of the Kil-0 early flowering locus. **(A)** Heatmap of differentially expressed genes in Kil-0 versus Col-0 in 21°C as analyzed by RNA-seq. The upper panel shows the fold change expression between Kil-0 and Col-0 of the seven expressed genes in the 31.3 kb mapping interval depicted in S2E Fig. The lower panel shows expression values of 267 flowering time genes. Fold changes are log_2_ transformed, blue represents upregulation and red downregulation in Kil-0 compared to Col-0. Significantly differentially (*p < 0*.*01*) expressed genes are marked with arrows. **(B)** Gene model of the *FLM* ^*Kil-0*^ locus of the Col-0 reference gene with the alternatively used exons 2 and 3 that give rise to *FLM-ß* and *FLM-δ* are shown in blue. Black boxes indicate exons, grey lines are 5’- or 3’-untranslated regions or introns. **(C)** Read coverage of the Kil-0 genomic data on the Col-0 reference gene model. Small sequence polymorphisms are indicated below the graph. Distribution of two independently assembled contigs from Kil-0 genomic sequence reads reveal the presence of a 5.7 kb insertion in *FLM* intron 1 with homology to the *A*. *thaliana* transposon At_LINE1-8. Sequence elements with homology to the LINE element are shown in dark blue in the lowermost panel. The respective positions of the primers used for the screening of further *A*. *thaliana* accessions are depicted.

The prominent early flowering of Kil-0 at 15°C (hitherto FT15) reliably allowed distinguishing between Kil-0 and Col-0. Analyses of F_1_ and F_2_ Kil-0 x Col-0 plants indicated that the Kil-0 flowering phenotype was determined by a major-effect recessive locus (Fig 1C, 1D and 1E). We subsequently mapped the FT15 locus to a 968 kb genomic region (S2A and S2B Fig). After selfing F_2_ plants with a recombination event in this interval, we identified 49 F_3_ plants with an early (Kil-0) and 41 F_3_ plants with a late (Col-0) flowering behavior (S2C and S2D Fig). We further narrowed down the interval of interest to a 151 kb region between 28.9 and 29.1 Mb on chromosome 1 (S2E Fig) and sequenced pools of 15 early and 9 late flowering F_3_ recombinants (S1 Table). Additionally, we sequenced the Kil-0 genome and identified 309 high confidence SNPs (single nucleotide polymorphisms) in the 151 kb mapping interval (S2E Fig). We smoothed the allele frequencies of the 309 SNPs of the two pools of early and late flowering F_3_ plants using LOESS (locally weighted scatterplot smoothing) and calculated the difference (Δ*f*) between them. A fraction of Δ*f* > 25% defined a final mapping interval of 31.3 kb that comprised eleven annotated genes (S2E Fig). Since the flowering phenotype of Kil-0 segregated in a recessive manner (Fig 1C), we assumed that a potential candidate gene might show reduced transcript abundance in comparison to Col-0. When we investigated RNA-seq data from 10 day-old Kil-0 and Col-0 plants grown at 21°C, we identified *FLOWERING LOCUS M* (*FLM*) as the only gene within the 31.3 kb region that was expressed at a significantly lower level in Kil-0 than in Col-0 (Fig 2A and S2 Table). *FLM* was also the most strongly downregulated gene in Kil-0 when we specifically analyzed 267 genes with a role in flowering time regulation (Fig 2A and S3 Table). These data suggested that *FLM* (*FLM* ^*Kil-0*^) may be causative for early flowering in Kil-0.

### Kil-0 *FLM* harbors a LINE retrotransposon

Since the gene expression analyses had indicated that *FLM* may be the causative locus for early flowering im Kil-0, we compared the *FLM* genomic loci from Kil-0 and Col-0 at the molecular level. We found, however, no SNPs in the *FLM* coding sequence and only a few SNPs in the *FLM* promoter or introns. Interestingly, read coverage was greatly reduced at the beginning of the first *FLM* intron in Kil-0 (Fig 2B and 2C). Since a structural polymorphism could cause such a read coverage reduction, we *de novo* assembled the Kil-0 genomic sequencing reads and identified an insertion in *FLM* ^*Kil-0*^ that was flanked by two contigs corresponding to two halves of the *FLM* ^*Col-0*^ locus at the predicted insertion site (Fig 2C). We PCR-amplified this region and confirmed the presence of a 5.7 kb insertion in the first intron of *FLM* ^*Kil-0*^ (Fig 2C). The inserted sequence showed similarity (68% identity, 86% coverage) to *A*. *thaliana* ATLINE1_8 (hereafter called LINE insert), a non-LTR (NON-LONG TERMINAL REPEAT LONG INTERSPERSED NUCLEAR ELEMENTS) retrotransposon with six typical retrotransposon domains as well as a perfect copy of the second exon of a *RIBONUCLEASE H-LIKE* (AT1G04625) gene from chromosome 1 of the Col-0 genome (S4 Table and Figs 2C and S3). We considered this *FLM* insertion polymorphism as the most likely cause for early flowering in Kil-0 and subsequently conducted genetic experiments to confirm this hypothesis.

### *FLM* ^*Kil-0*^ is a functional temperature-sensitive *FLM* allele

**Fig 3:**
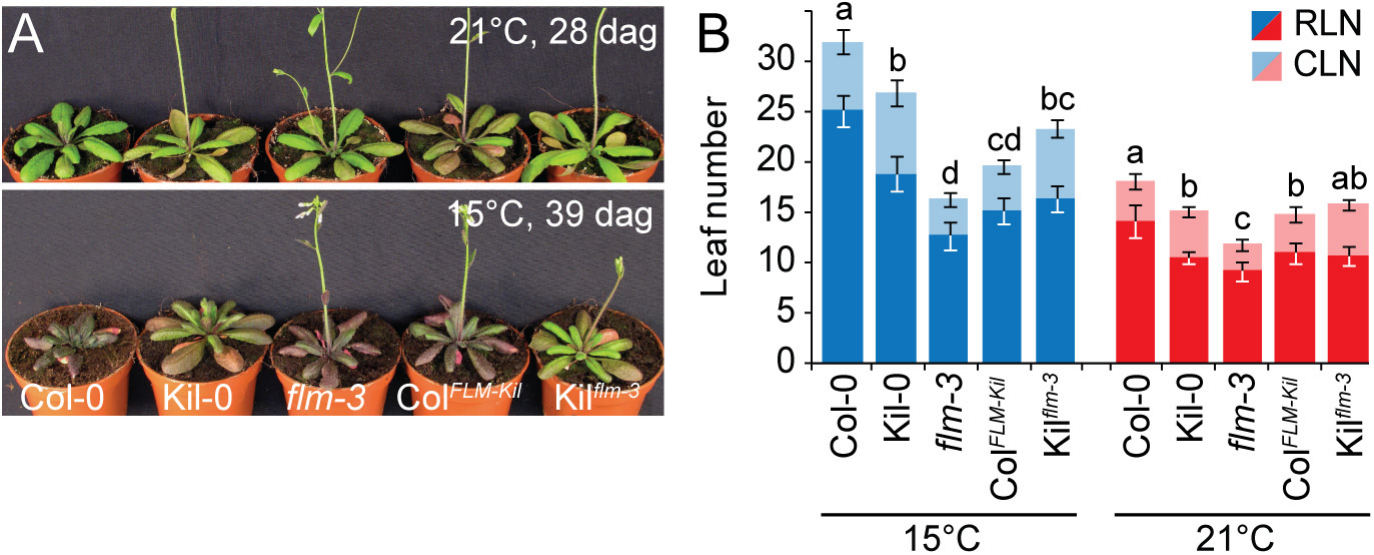
*FLM* ^*Kil-0*^ accelerates flowering. **(A**) Photographs of representative 28 and 39 day-old plants of the Col^*FLM-Kil*^ and Kil^*flm-3*^ backcross population in comparison to Col-0, Kil-0, and *flm-3* grown at 15°C and 21°C in long day photoperiod. **(B)** Averages ± SD of the quantitative flowering time analysis (RLN, CLN; rosette and cauline leaf number) of the genotypes shown in (A). Similar letters indicate no significant difference of total leaf number (Tukey HSD, *p < 0*.*05*).

Loss of *FLM* results in early flowering in the *flm-3* allele in 23°C-grown Col-0 [17, 18]. To be able to compare the effect of the *FLM* ^*Kil-0*^ locus with that of *FLM* ^*Col-0*^ and the *flm-*3 loss-of-function allele, we backcrossed *FLM* ^*Kil-0*^ six times into Col-0 to establish Col^*FLM-Kil*^. Col^*FLM-Kil*^ flowered much earlier than Col-0 but not as early as the *flm-3* knock-out mutant (Fig 3). We also introgressed *flm-3* with four backcrosses into Kil-0 to obtain Kil^*flm-3*^. This introgression line flowered earlier than Kil-0. Although we performed marker assisted backcrossing, we cannot exclude the possibility that a background effect has an additional influence on flowering time regulation in the two described backcross lines. However, since the results between the two backcross lines are consistent with their effects in the original backgrounds, we considered it highly likely that the genetic data reflect *FLM* activity and concluded that the activity of *FLM* ^*Kil-0*^ was intermediate between that of a functional *FLM* ^*Col-0*^ allele and the *flm-3* loss-of-function allele (Fig 3).

The phenotypic differences between Col-0 and Col^*FLM-Kil*^ as well as those between Col-0 and *flm-3* were more pronounced at 15°C than at 21°C (Fig 3). This was in agreement with our analysis of *flm-3* in a range of growth temperatures, which had revealed that *FLM* makes a particularly prominent contribution to flowering time control at 15°C (S4 Fig). Since 15°C is closer to the average temperature in the native range of the species than the commonly used 21°C, the strong effect of *FLM* on flowering at 15°C should be considered a physiologically and ecologically relevant phenotype [11, 27].

### Gene expression and alternative splicing of *FLM* are differentially regulated in Kil-0

**Fig 4:**
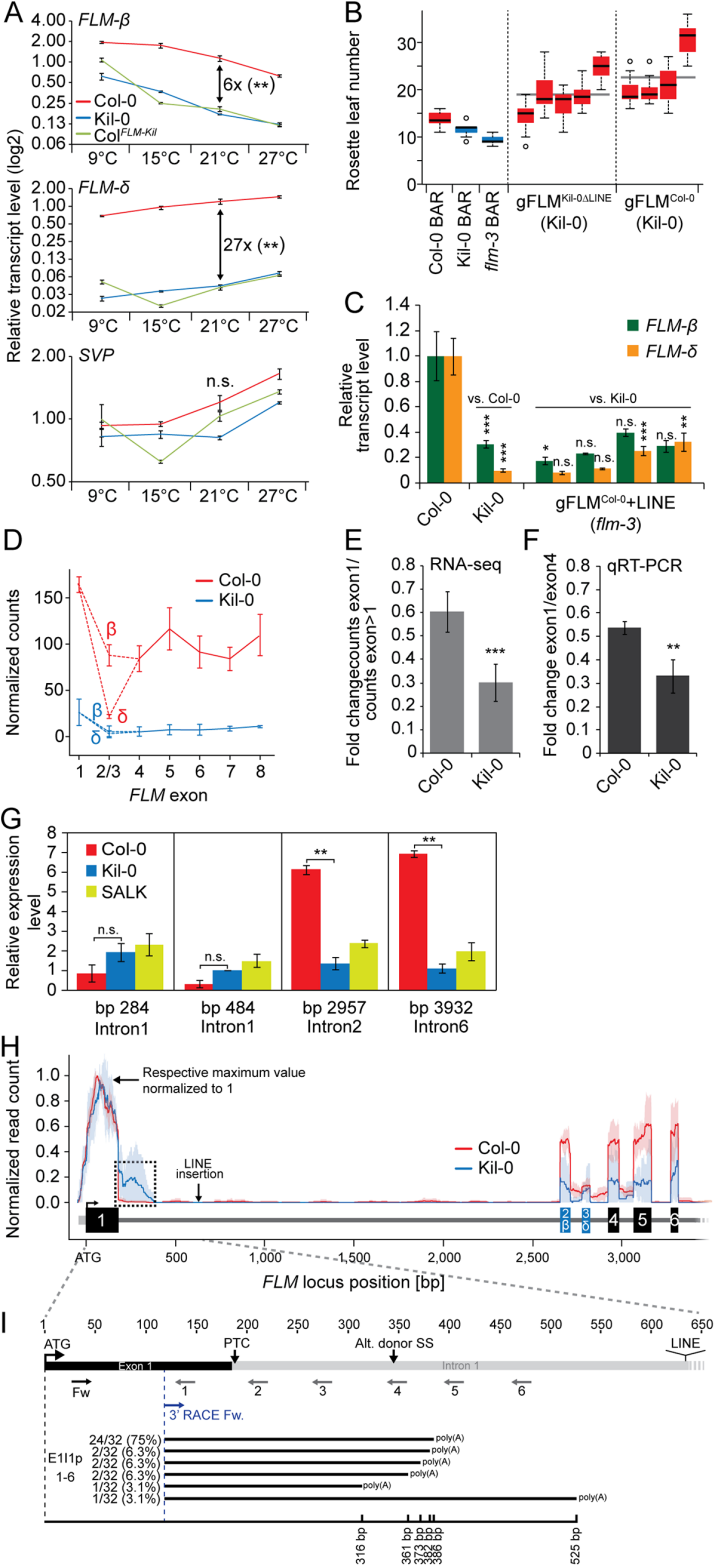
The large insertion in the first intron affects splicing efficiency and gene expression of *FLM*. **(A)** qRT-PCR analysis of *FLM-ß*, *FLM-δ*, and *SVP* expression in ten day-old Col-0, Kil-0, and Col^*FLM-Kil*^ plants. Seven day-old seedlings grown at 21°C were transferred to the indicated temperature and grown for further three days. The fold change differences between Col-0 and Kil-0 at 21°C are indicated in the graph. Error bars represent SE of three biological replicates. **(B)** Quantitative flowering time analysis of four—five independent T_2_ lines in the Kil-0 background that are either hemi- or homozygous for the respective transgene construct. 14–20 transgenic plants grown at 21°C under long day photoperiod were analyzed for each genotype. Transgenic T_2_ lines expressing the empty pGREEN0229 vector control construct (BAR+) in the Col-0, Kil-0, and *flm-3* lines were analyzed for comparison. Outliers were determined based on 1.5 x IQR (interquartile range). **(C)** qRT-PCR analysis of *FLM-ß and FLM-δ* expression of four independent homozygous T_3_ lines of the indicated construct in the *flm-3* background. Plants were grown for ten days under 21°C long day photoperiod. Error bars represent SE of three biological replicates. **(D)** Graph displaying normalized read counts for the seven *FLM* exons in Col-0 and Kil-0, including the differentially spliced exon 2 that gives rise to the *FLM-ß* and *FLM-δ* isoforms. **(E)** Quantification of the normalized read counts shown in (D). The average ratios and SD from three biological replicates of the read counts of exon 1 compared to the read counts of exon 2 to exon 7 are shown. **(F)** qRT-PCR-based verification of the RNA-seq result shown in (E) using fragments corresponding to *FLM* exon 1 and exon 4. The ratios ± SE of the respective expression values are shown from three biological replicates. **(G)** qRT-PCR quantification of unspliced pre-mRNA from isolated nuclei. Primers are located in *FLM* introns 1, 2, and 6 and *ACT8* introns 2 and 3, respectively. Primer sequences are provided in S7 Table. The position of the respective reverse primer relative to the ATG start codon is indicated. Fold changes are averages ± SE of two biological replicates are indicated. Student’s t-tests: ** = p ≤ 0.01; n.s., not significant. **(H)** Normalized read counts for *FLM* as detected by RNA-seq in Col-0 and Kil-0 mapped to the genomic *FLM* locus from exon 1 through exon 5. For both accessions, the number of mapped RNA-seq reads was normalized to range from 0 (no expression) to 1 (maximum expression). Lines indicate the mean expression level and light blue and light red areas indicate the 5% and 95% confidence intervals that were determined across the biological replicates of one genotype. Exons are represented as boxes, untranslated regions and introns as lines. Note the relative increase in intron 1 reads in Kil-0 as indicated with a dotted line. The position of the LINE insertion in *FLM* ^*Kil-0*^ is indicated by an arrow. Student’s t-tests were performed as indicated: * = p ≤ 0.05; ** ≤ 0.01; *** = p ≤ 0.001; n.s., not significant. **(I)** Schematic representation of the primers used for the qRT-PCR quantification of *FLM* intron 1 sequence-containing transcripts and the 3’-RACE PCR as indicated by arrows below the gene model; PTC, premature termination codon. A schematic representation of the intron 1 sequence-containing polyadenylated transcripts is indicated in the lower part of the panel. Numbers on the left indicate the frequency of each transcript among the 32 individual sequenced clones. The black line indicates the length of each transcript respective to the ATG start codon.

*FLM* produces two major splice isoforms, *FLM-ß* and *FLM-δ*, in the Col-0 accession [19, 20]. *FLM-ß* is the predominant splice form in cooler and *FLM-δ* is the predominant splice form in warmer temperatures [19]. Since the *FLM* ^*Kil-0*^ allele had weaker effects on flowering time than the *flm-3* loss-of-function allele and since *FLM* ^*Kil-0*^ did not have polymorphisms in the *FLM* coding region, we hypothesized that the temperature-sensitive flowering of Kil-0 may be caused by changes in *FLM* expression or *FLM* alternative splicing. To examine this, we transferred seven day-old 21°C-grown Col-0 and Kil-0 plants for three days to 9°C, 15°C, 21°C or 27°C and examined the effects on *FLM-ß* and *FLM-δ* transcript abundance. In agreement with published data, we observed a respective decrease of *FLM-ß* and an increase of *FLM-δ* in response to warmer temperatures (Fig 4A) [19, 20]. Importantly, temperature-dependent changes in the abundance of the *FLM* isoforms were maintained in Kil-0 as well as in Col^*FLM-Kil*^ but the overall *FLM* transcript abundance was strongly reduced compared to Col-0. For example, when comparing the values at 21°C, Kil-0 and Col^*FLM-Kil*^ had six-times less *FLM-ß* and 27-times less *FLM-δ* than Col-0 (Fig 4A).

It was previously shown that *FLM-ß* represses and that *FLM-δ* promotes flowering at 16°C when introduced as transgenes in the *flm-3* background [19–21]. It was further proposed that FLM-ß forms heterodimers with SVP (SHORT VEGETATIVE PHASE) to prevent flowering by direct DNA-binding to repress the transcription of *FT* and *SOC1*. Conversely, *FLM-δ* would form inactive heterodimers with SVP and would thereby indirectly induce flowering by relieving the repression from *FT* and *SOC1*. To understand the effects of this differential regulation, we measured *SVP*, *FT*, and *SOC1* expression levels. Whereas *SVP* expression was similar between the different genotypes, *FT* and *SOC1* were expressed more strongly in the early flowering Kil-0 or Col^*FLM-Kil*^ than in Col-0 (Figs 4A and S5). We thus concluded that the reduced expression of *FLM* was likely the cause for the early flowering of Kil-0 and Col^*FLM-Kil*^ and that the temperature-dependent differential accumulation of *FLM-ß and FLM-δ* could be the basis of the temperature-sensitive flowering time in Kil-0. Since Kil-0 flowered earlier than Col-0, we assumed that the effect of the downregulation of the repressive *FLM-ß* isoform was dominant over the downregulation of the flowering activating isoform *FLM-δ*.

### The LINE insertion affects *FLM* splice isoform abundance

To test whether the large insertion in *FLM* ^*Kil-0*^ was the causative polymorphism for low *FLM* expression and the specific reduction in the *FLM-ß* isoform, we transformed Kil-0 with a genomic fragment of *FLM* ^*Col-0*^ (including 2 kb promoter plus 5'-UTR [untranslated region] and 0.5 kb 3'-UTR sequence) as well as an *FLM* ^*Kil-0*^ genomic variant with an engineered deletion of the LINE-insertion (*FLM* ^*Kil-0ΔLINE*^). We found that *FLM* ^*Col-0*^ as well as *FLM* ^*Kil-0ΔLINE*^ delayed flowering in Kil-0 (Fig 4B). We also tested whether the 5.7 kb insertion had a comparable effect on *FLM* transcript abundance when engineered into the *FLM* ^*Col-0*^ reference and introduced a *FLM* ^*Col-0*^ transgene with an engineered 5.7 kb LINE insertion into the *flm-3* loss-of-function mutant. Indeed, the LINE insertion reduced *FLM* expression and changed *FLM* splicing, similar to the *FLM* ^*Kil-0*^ allele (Fig 4C). We thus considered it very likely that the LINE insertion in the first intron of *FLM* was the causative polymorphism for reduced *FLM* transcript abundance, differential *FLM* splice isoform accumulation, and early flowering in Kil-0.

To gain an understanding of the molecular effect of the LINE insertion on *FLM* transcription and splicing, we compared the exon usage of Col-0 and Kil-0 using RNA-seq data. In Col-0, we detected a strong differential use of alternative exons 2 (*FLM-ß)* and 3 (*FLM-δ*) that define the two dominant *FLM* isoforms (Fig 4D), with the *FLM-ß*-specific exon 2 being more abundant than the *FLM-δ*-specific exon 3. In Kil-0, we detected generally fewer reads for all exons including exon 1, indicating that the LINE insertion between exon 1 and exon 2 controls the overall *FLM* transcript abundance (Fig 4D). Furthermore, we noted that the read coverage of the exons following intron 1, including the alternatively used exons 2 (ß) and 3 (δ) was more strongly reduced in Kil-0 than in Col-0, suggesting that the inserted LINE element may also negatively control *FLM* splicing efficiency (Fig 4D, 4E and 4F). To approximate the rate of *FLM* transcription, we determined the levels of unspliced *FLM* pre-mRNA using primers located in intron 1 (upstream of the LINE insertion), and intron 2 and 6. No significant differences in pre-mRNA levels were detected when we amplified intron 1 indicating a comparable transcription rate between the two accessions. However, the relative abundance of intron 2 and intron 6, which are located downstream from the insertion was strongly reduced in Kil-0, indicative for a partial premature termination of *FLM* transcription (Figs 4G, S6A and S6B). We next mapped the RNA-seq reads from Col-0 and Kil-0 to the *FLM* ^*Col-0*^ genomic locus paying particular attention to the exon-intron junctions (Fig 4H). Whereas the read coverage dropped sharply at the exon 1-intron 1 junction in Col-0, reads covering the beginning of intron 1 could be readily retrieved in Kil-0 (Fig 4H). This finding suggested that the LINE insertion resulted in aberrant splicing within Kil-0 intron 1. This change in the splicing pattern had an impact on the abundance of the *FLM-ß* and *FLM-δ* full-length transcripts but, importantly, the respective full-length mRNAs were still generated as confirmed by semi-quantitative RT-PCR (S6C Fig). We then used qRT-PCR with primers spanning only exon 1 or exon 1 and parts of intron 1 to validate the occurrence of transcripts containing intron 1 sequences (Fig 4I). In Col-0 as well as in Kil-0, we found transcripts including intron 1 sequences until 350 bp from the start codon (Figs 4I and S6D). The abundance of intron 1 sequence-containing reads was much higher in Kil-0 than in Col-0 indicating that the insertion may indeed promote premature transcription termination possibly in combination with aberrant splicing. Since transposon insertions were reported to induce alternative polyadenylation [28–30], we examined whether the corresponding intron 1 sequence-containing transcripts were polyadenylated and performed 3’-RACE (rapid amplification of cDNA ends) experiments. The RACE PCR yielded two abundant fragments, one corresponding in size to the full length transcript and one smaller fragment that was much more abundant in Kil-0 than in Col-0 (S6E Fig). We cloned and sequenced products and determined six different polyadenylated transcripts containing intron 1 sequences. One of these fragments represented the most abundant species (75%) among the 32 independent sequences (Fig 4I). Premature translation termination codons (PTC) located distantly from splice sites frequently trigger the degradation of aberrant transcripts through the NMD- (non-sense mediated decay-) pathway [31, 32]. We identified a PTC in intron 1, just two bases downstream from the exon1-intron1 border and hypothesized that the aberrant FLM^Kil-0^ transcripts may be NMD targets (Fig 4I). When we used the translation inhibitor CHX (cycloheximide) to mimic the molecular phenotype of NMD-defective mutants [32, 33], we detected indeed a significant increase in the abundance of two aberrant transcripts containing intron 1 sequences (S6F and S6G Fig). In summary, we concluded that the early flowering of Kil-0 correlated with the presence of a LINE insertion in *FLM* intron 1. Further, the LINE insertion did not affect *de novo* FLM transcription initiation but partially impaired the formation of full length transcripts, possibly as a result of premature transcription termination and the formation of aberrantly spliced polyadenylated transcripts that are targeted for NMD. Alternative molecular mechanisms that were not examined here may of course also be suitable to explain the overall reduction in *FLM* transcript abundance in Kil-0.

### The geographic distribution of the *FLM* ^*Kil-0*^ allele is consistent with a recent adaptive selective sweep

**Fig 5:**
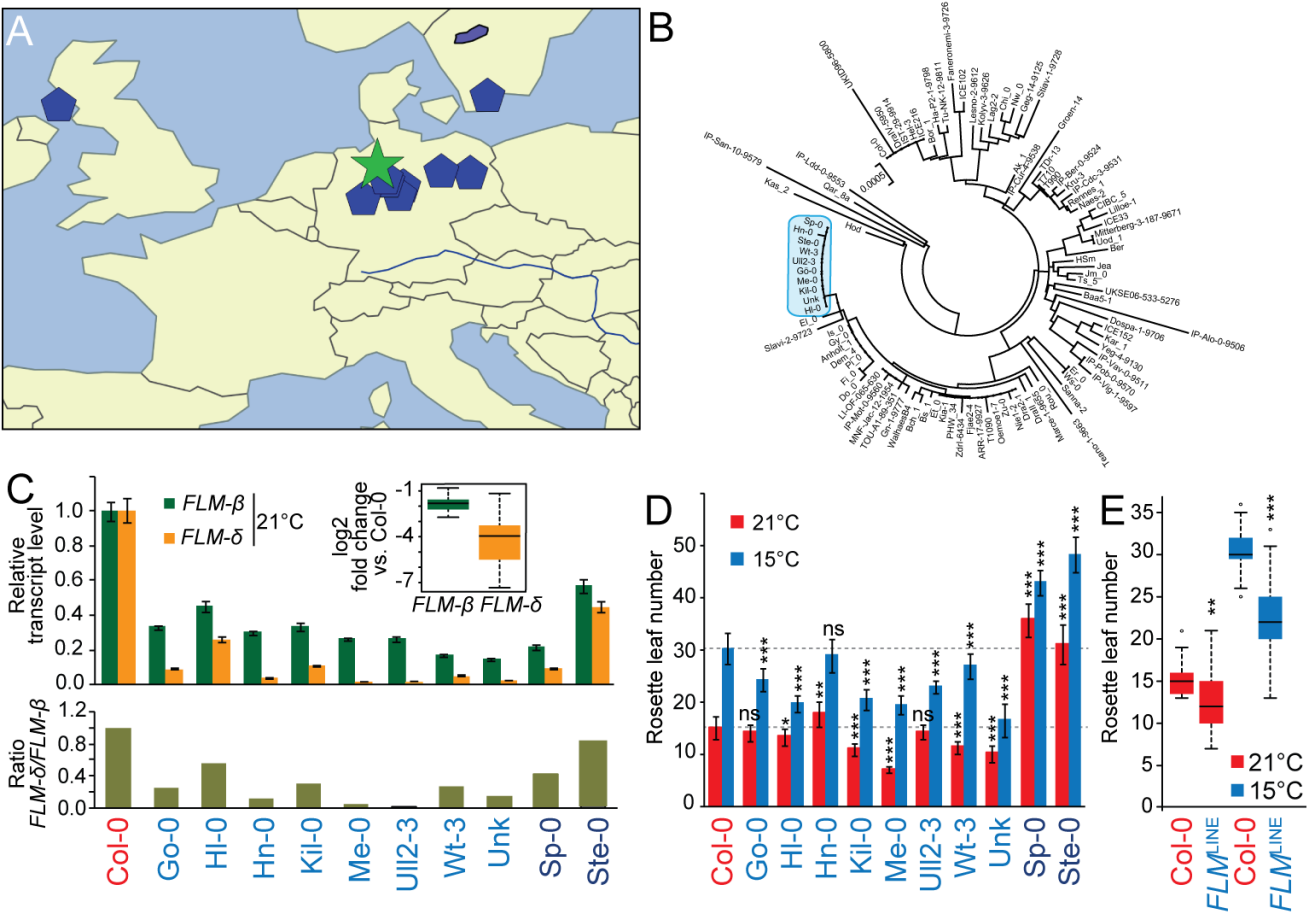
Geographical distribution and molecular analysis of *FLM* ^*LINE*^ accessions. **(A)** Geographical distribution of the *FLM* ^*LINE*^ accessions (blue pentagons) with the centroid (green star). Please note that one the accession with an ambiguous name was excluded due to the unavailability of geographic information. **(B)** Neighbour-Joining tree showing the genetic relationship among *FLM* sequences. The *FLM* sequences of the 10 *FLM* ^*LINE*^ accessions were analyzed together with Col-0 and further 88 randomly selected sequences. The clade harboring all *FLM* ^*LINE*^ alleles is marked in blue; see S8 Fig for a detailed representation of the entire tree. **(C)** qRT-PCR analyses of *FLM-ß and FLM-δ* transcript abundance of ten day-old seedlings of all *FLM* ^*LINE*^ accessions when grown in 21°C long day photoperiod (upper panel). Averages ± SE of three measurements after normalization to Col-0 as a control are shown. The inserted graph shows the summarized log_2_-transformed fold changes of *FLM* ^*LINE*^ accessions only (Col-0 excluded). The lower panel displays the ratios of *FLM-δ over FLM-ß* when normalized to Col-0. **(D)** Quantitative flowering time analysis of *FLM* ^*LINE*^ accessions grown at 21°C and 15°C under long day photoperiod. Dashed lines mark the respective values for Col-0. Please note that Sp-0 and Ste-0 are vernalization-sensitive accessions. **(E)** The right graph shows the summarized rosette leaf number of the eight vernalization-insensitive *FLM* ^*LINE*^ accessions. Student’s t-tests were performed in comparison to Col-0 unless indicated otherwise: * = p ≤ 0.05; ** ≤ 0.01; *** = p ≤ 0.001; n.s., not significant. El-0 was identified relatively late in this study and not included in this detailed analysis.

To gain information about the distribution of the *FLM* ^*Kil-0*^ structural polymorphism across the native range of the species, we screened a genetically highly diverse set of accessions from a previously published HapMap population, which we supplemented with selected laboratory accessions to obtain a final population with 419 accessions (S6 Table and Figs 2E and S7) [34]. Through PCR-based screening, we identified nine additional accessions with a LINE insertion in the same position as in Kil-0 from Scotland (Kil-0) and Sweden (Ull2-3) to Germany (8 accessions) (S5 Table and Fig 5A and 5B). We subsequently also analyzed genome sequences from 1128 *A*. *thaliana* accessions (www.1001genomes.org) and confirmed by this approach seven *FLM* ^*LINE*^ accessions and identified El-0 as an additional *FLM* ^*LINE*^ accession from Germany (S7 Fig).

Sequence analyses of the LINE insertions revealed a high sequence similarity between the ten *FLM* ^*LINE*^-accessions (S9 Fig). Taking into account a spontaneous mutation rate of 6 to 7×10^−9^ per site per generation, approximately one to three seed generations per year, and the absence of any selective pressure on the insertion, we calculated that the common ancestor probably originated only 8.000 to 30.000 years ago [35, 36]. We found that the *FLM* ^*LINE*^ accessions belonged to genetically differentiated clades and were thus truly independent [37]. Additionally we performed a phylogenetic analysis using the genomic sequence of the *FLM* locus of these ten *FLM* ^*LINE*^ accessions together with Col-0 and 88 randomly selected accessions, which revealed that the *FLM* ^*LINE*^ lines clustered into one clade when the *FLM* locus was analyzed in isolation (Figs 5B and S8).

Similarly to Kil-0, all *FLM* ^*LINE*^-accessions had low expression of the *FLM-ß* and *FLM-δ* isoforms (Fig 5C). Apart from two late-flowering vernalization-dependent accessions (Sp-0 and Ste-0), seven of the remaining eight *FLM* ^*LINE*^ accessions flowered earlier than Col-0 at 15°C (Figs 5D, S9A and S9B) [12, 26]. Consistent with the fact that these accessions come from genetically highly diverse groups, these lines showed a substantial variation in flowering time between them. However, when flowering time data of the vernalization-independent *FLM* ^*LINE*^ accessions was averaged, we measured a significant reduction in flowering time at 21°C and 15°C in comparison to Col-0 (Fig 5E). Thus, the LINE insertion correlated with early flowering in summer annual accessions. Our data thus suggests that the *FLM* ^*Kil-0*^ allele arose comparatively recently and subsequently spread geographically to contribute to flowering time regulation in a background- and temperature-dependent manner.

When we analyzed the ten *FLM* ^*LINE*^-accessions for *FLM* expression by qRT-PCR, we noted a prominent variation in the abundance of the *FLM-δ* isoform but relatively stable expression levels of *FLM-ß* (Fig 5C). We therefore asked whether *FLM* polymorphisms other than the LINE insertion could explain these differences. However, all ten *FLM* ^*LINE*^ accessions were highly similar in this region and we identified only four different additional polymorphic sites (S9C Fig). Since a Mann-Whitney test (p > 0.05) indicated that none of these polymorphisms was significantly associated with *FLM-ß* and *FLM-δ* abundance or the ratio between these two isoforms, we concluded that the regulation of the *FLM-ß* and *FLM-δ* transcript abundance may be regulated in *trans*.

### The first intron carries important regions for isoform-specific *FLM* regulation

Specifically within the family of MADS-box transcription factor genes, various cases are known where structural polymorphisms within the first intron enhance or repress gene expression [10, 38–41]. To test if *FLM* intronic sequences contributed to *FLM* expression, we transformed *flm-3* mutants with constructs for the expression of the *FLM-ß* or *FLM-δ* coding sequences under control of a 2.1 kb *FLM* promoter fragment (S10 Fig). Importantly, none of the resulting *FLM-ß* or *FLM-δ* T_1_ transformants expressed significant levels of the respective transgene or showed a suppression of the *flm-3* early flowering phenotype (S10 Fig). Since *FLM* was expressed from corresponding genomic constructs containing all introns and since it was previously shown that *FLM* is expressed from the above-described construct when only intron 1 is included [19], we concluded that intron 1 was essential for *FLM* expression.

**Fig 6:**
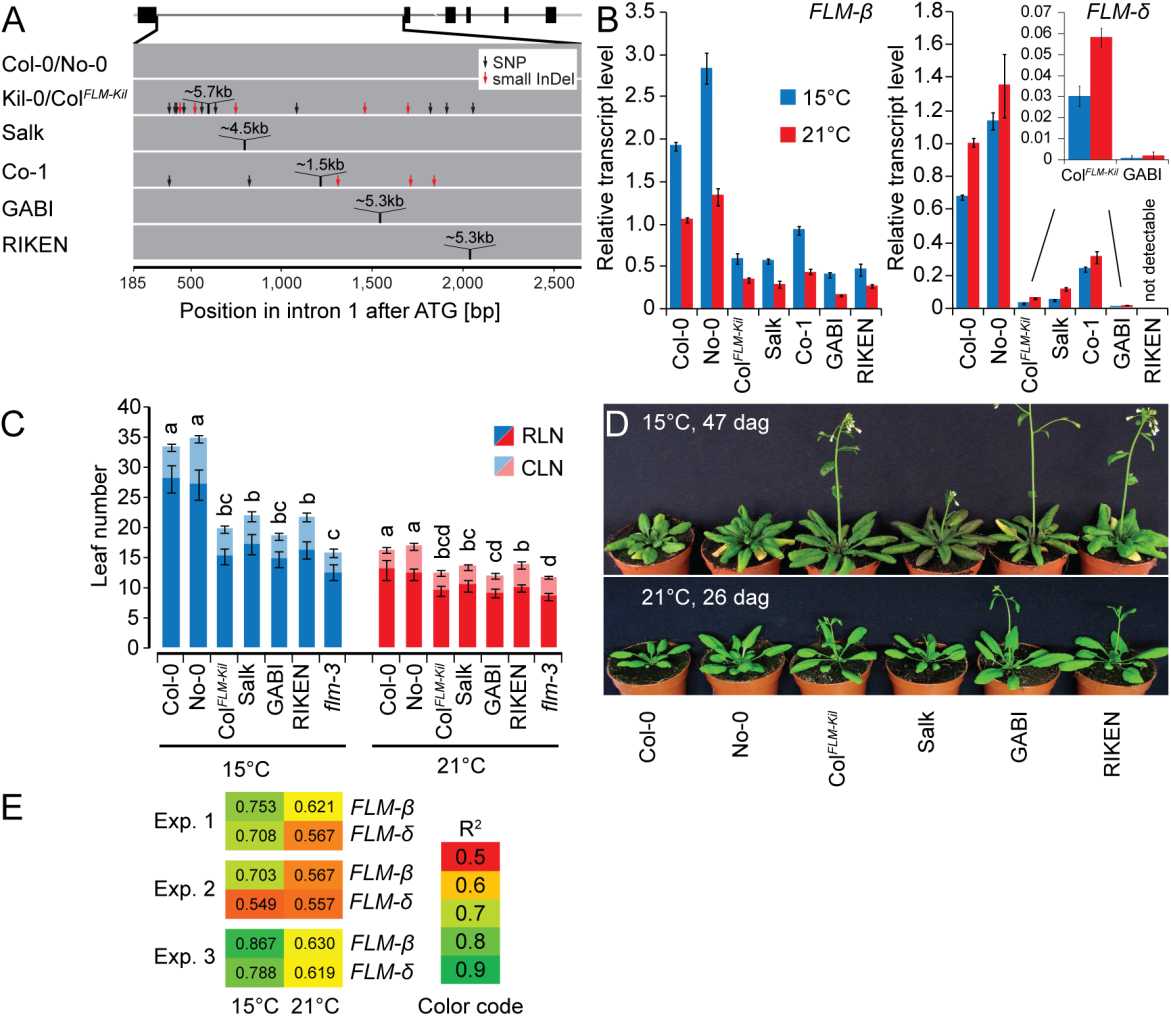
The first intron carries important regions for isoform-specific *FLM* abundance. **(A)** Schematic representation of the insertion lines used in this study. The *FLM-ß* gene model is depicted as shown in Fig 3C, intron 1 of the respective lines is depicted as a grey bar. **(B)** *FLM-ß* and *FLM-δ* expression of the lines shown in (A). Seven day-old seedlings were grown at 21°C and then transferred to 21°C or 15°C for further three days. Shown are averages and SE of three biological replicates. **(C)** Quantitative flowering time analysis of the lines shown in (A) with Col-0 and No-0 as wild type controls. Similar letters indicate no significant difference of total leaf number (Tukey HSD, *p < 0*.*05*). **(D)** Representative photographs of the lines shown in (A) at 26 and 47 days after germination (dag) when grown at 21°C and 15°C temperature and under long day photoperiod. **(E)** Correlation analysis of the flowering time experiment shown in Fig 6C and further two independent experiments (15°C and 21°C) with the expression values shown in Fig 6B. Note that flowering time values of the loss-of-function allele *flm-3* were also included in this analysis but that its *FLM* expression was set to 0 (no expression). R^2^ values from linear regression are shown.

To address whether sequence identity, sequence length or the specific insertion site within intron 1 conferred the effect of the LINE element on *FLM* expression and splicing, we examined the effects of T-DNA or DS (Dissociator) transposon element insertions in *FLM* intron 1 [42–44]. These intron 1 insertions were of similar size (4.5 kb to 5.3 kb) to the 5.7 kb LINE insertion and present in the Col-0 and No-0 (Nossen-0) accessions: Salk_068360 (Salk, Col-0), RATM13-4593-1 (RIKEN, No-0) and GK_487H01 (GABI, Col-0) (Fig 6A). Additionally, we included Co-1 that carries a ~1.5 kb insertion in *FLM* intron 1 (Fig 6A). When we determined the effects of these structural variants on *FLM* transcript accumulation and alternative splicing in plants grown at 15°C and 21°C, we found that the insertion correlated in each case with reduced *FLM* transcript abundance when compared to the controls (Fig 6B). Furthermore, *FLM-δ* was in each case more strongly reduced than *FLM-ß*, inviting the conclusion that increases in intron length, regardless of the molecular identities of the insertions, resulted in decreased *FLM-ß* and *FLM-δ* expression and changes in the ratio between the isoforms. This was further confirmed by the molecular analysis of pre-mRNA transcript abundance, the formation of aberrant polyadenylated transcripts and transcript targeting to the NMD pathway, which we performed in parallel for the Salk insertion line, Col-0, and Kil-0 (Figs 4G and S6). In this analysis, we identified in each case the same molecular defects in the Salk insertion line as in Kil-0. At the same time, we noted that the temperature-sensitive regulation of the *FLM* isoforms was maintained in all lines. Interestingly, insertions in the second half of the intron as present in the GABI and RIKEN lines caused a particularly strong reduction in the expression of *FLM-δ* expression (Fig 6B). We thus concluded that insertions in the second half of the intron may have additional effects on *FLM-δ* splicing.

To examine the phenotypic consequences of the observed transcriptional changes of the *FLM* insertion lines, we evaluated their flowering at 15°C and 21°C. We thereby focused on the Salk and Col^*FLM-Kil*^ as well as the GABI and RIKEN lines, which had contrasting phenotypes with regard to the abundance of the *FLM-δ* isoform. Regardless of the differences in expression of the *FLM-δ* isoform, all lines flowered earlier than the respective wild type, and this effect was particularly prominent at 15°C (Fig 6C and 6D). Importantly, we did not notice any pleiotropic effects on plant growth or plant height for the tested alleles suggesting that *FLM* acts specifically on flowering time regulation (Figs 6D and S11). We next evaluated to what extent changes in *FLM-ß* or *FLM-δ* abundance could explain the observed difference in flowering time. To this end, we correlated datasets on flowering time and *FLM* expression from three independent flowering time experiments (Fig 6E). In each experiment, expression of *FLM-ß* correlated much better with flowering time than *FLM-δ* and this effect was particularly pronounced at 15°C (Fig 6E). Thus, *FLM-ß* expression levels alone rather than the ratio between *FLM-ß* and *FLM-δ* have a prominent effect on flowering time regulation in *A*. *thaliana*.

## Discussion

**Fig 7:**
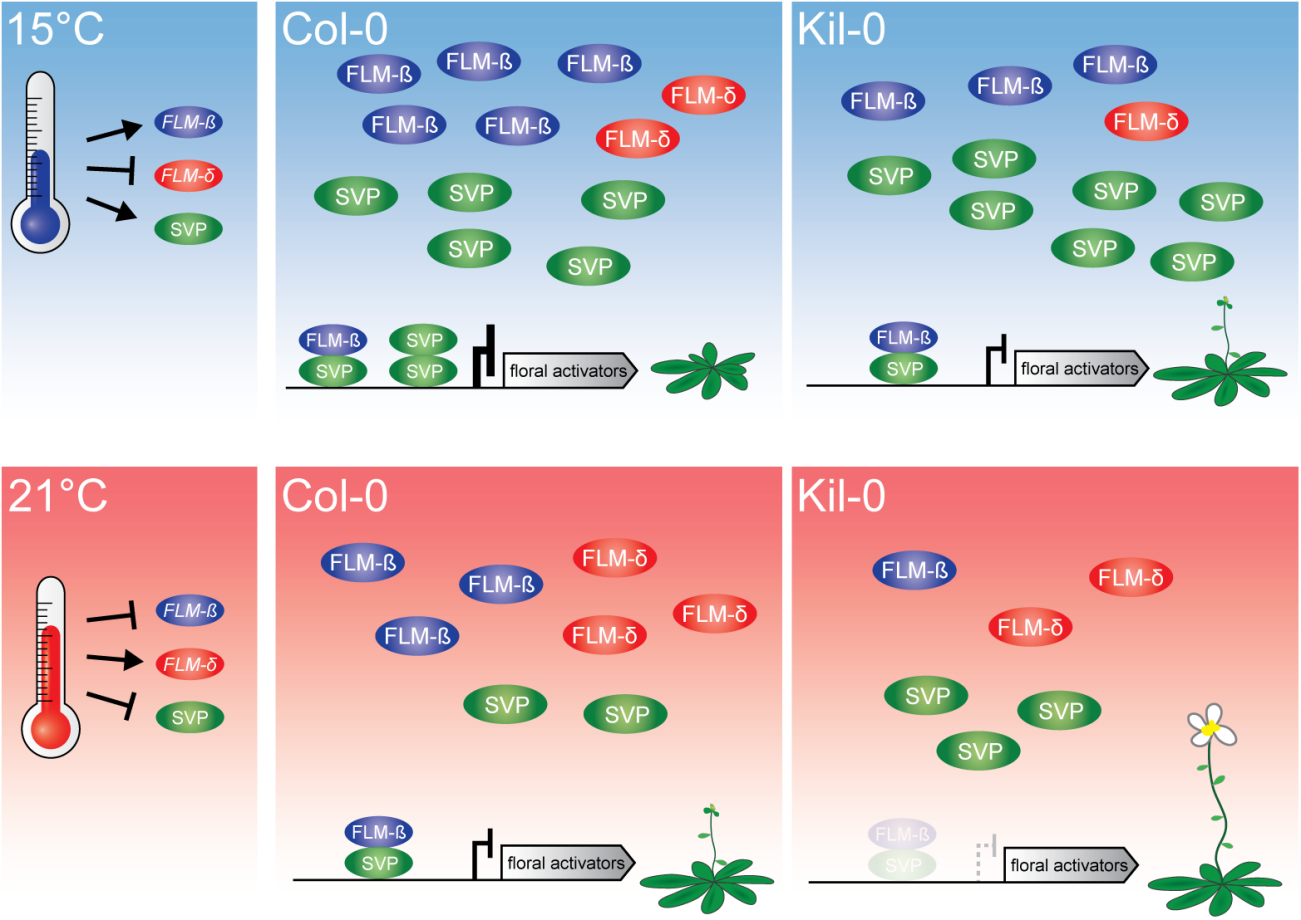
Model of the proposed mode of FLM action. At 15°C, flowering is delayed due to an active repression of floral activators by FLM-ß-SVP heterodimers. An increased ambient temperature (21°C) results in decreased levels of the transcriptional repressive FLM-ß-SVP protein complexes and floral activator genes are expressed. Flowering in Kil-0 (right) might be accelerated in comparison to Col-0 due to a decreased abundance of FLM-ß at 15°C as well as at 21°C while *SVP* is unaltered in the different temperatures and between Kil-0 and Col-0. Note that our study suggests a minor role of FLM-δ in the control of flowering time in the lines and conditions examined. The present model assumes that protein abundance follows transcript abundance except for SVP, which is degraded in response to increasing temperature [20].

We have identified a recently evolved *FLM* allele from the accession Kil-0. The insertion of a LINE element in intron 1 of *FLM* ^*Kil-0*^ resulted in reduced *FLM* transcript abundance and correlated with an overall acceleration of flowering time that was particularly prominent at 15°C (Fig 7). We identified additional nine *FLM* ^*LINE*^ accessions that mainly represented lines collected from Germany. Although these *FLM* ^*LINE*^ accessions were highly homologous over the *FLM* locus, they represented accessions from genetically different clades indicating that *FLM* ^*LINE*^ was involved in recent adaptation to early flowering and that its rather narrow geographical distribution is likely due to the young demographic history of this allele.

The LINE element insertion of *FLM* ^*LINE*^ shares 68% homology with LINE class I retrotransposons from *A*. *thaliana*. Transposable elements are typically suppressed by epigenetic mechanisms and this suppression can also negatively interfere with the expression of neighboring genes [45–48]. Epigenetic regulation is also known to control the expression of MADS-box transcription factor genes. For example, the chromatin of *FLC* is modified during vernalization by lysine 27 methylation of histone 3 (H3K27me), a repressive mark, and several natural variants interfere with regulatory regions in the *FLC* intron 1 [41, 49–53]. Thus, the LINE insertion could interfere with the direct transcriptional regulation but, alternatively, also with the epigenetic control of the *FLM* ^*Kil-0*^ locus. However, previous genome-wide studies failed to identify epigenetic marks such as H3K27me on intron 1 of FLM [54] and we detected no differences in transcription rate between the insertion lines and the Col-0 reference. Furthermore, transposon and T-DNA insertions were reported to mediate alternative polyadenylation through the utilization of alternative polyadenylation sites [28–30]. Although we detected a higher abundance of short aberrant polyadenylated transcripts in the insertion lines, we consider it unlikely that the insertion itself provides *cis* elements that result in their synthesis. Aberrrant transcripts did not extend into the insertion and no differences in quantity and composition of these transcripts were detected between Kil-0 and the Salk line, which have molecularly distinct insertions. We concluded that the reduction of *FLM* full-length transcript abundance in Kil-0 is caused by a combination of partial premature termination of transcription and aberrant splicing due to the enlargement of the first intron.

Conversely, through experiments with *FLM* transgenes where intronic sequences were deleted, we could conclude that intron 1 was strictly required for *FLM* expression and activity. A contribution of intronic sequences in gene expression regulation, generally referred to as IME (intron-mediated enhancement), was previously reported for many genes, and in plants specifically for members of the MADS-box transcription factor family [55, 56]. Several studies have already identified corresponding intronic *cis*-regulatory elements, e.g. in intronic regions of the floral homeotic genes *AG* (*AGAMOUS*) and members of the *AGL6* (*AGAMOUS-LIKE6*)*-*subfamily [38–40, 57]. Several independent structural intron polymorphisms were also reported for the MADS-box factor and flowering time regulator *VRN1* (*VERNALIZATION1*) from wheat and barley. There, these structural differences in intron 1 composition can promote high *VRN1* expression and these differences are the main molecular cause for vernalization-independent flowering in many spring barley and wheat cultivars [10]. Thus structural intron polymorphisms, e.g. through transposon insertions, are a recurrent theme in the expression control of MADS-box transcription factors and the adaptation to the environment through these factors.

Interestingly, our expression analysis showed that the expression of the two *FLM* isoforms, *FLM-ß* and *FLM-δ* were differentially affected by the intron 1 insertions. Since this behavior was found in several accessions and was recapitulated by inserting a LINE-bearing *FLM* transgene in the Col-0 *FLM* allele, we judge that this regulation again is not related to the nature of the insertion in *FLM* but rather to the position of the insertion or the corresponding increase in intron length. It remains to be investigated, however, what the underlying molecular basis of the differential effect of the insertions on *FLM-ß* and *FLM-δ* abundance is.

Through investigations of insertion lines from different sources and ecotypes, we found that the position of the inserted sequence affected the relative abundance of *FLM-ß* and *FLM-δ*. In combination, the availability of these lines allowed the examination of flowering time and its correlation with the abundance of the two *FLM* splice variants.

Importantly, our study did not support a role for *FLM-δ* in flowering time control but rather suggests that *FLM-ß* abundance alone is the predominant determinant of flowering time in natural variants under the ecologically relevant temperature of 15°C. Thus, previous experiments exploring the contribution of *FLM-ß* and *FLM-δ* to flowering time control using transgenic approaches may have overestimated the contribution of *FLM-δ*, at least in the diverse genetic material and at the temperatures used in our study. At the same time, it cannot be ruled out that *FLM-δ* levels reached a subcritical level in the insertion lines analyzed in our study so that the contribution of *FLM-δ* could not be accurately determined. Along these lines, a recent study showed no contribution of the *FLM* locus to flowering time regulation in a large set of wild *A*. *thaliana* ectoypes in warm (27°C) temperature where *FLM-δ* is upregulated [58] indicating that even under temperature conditions that promote *FLM-δ* abundance no important regulatory role could be ascribed to it. Once a detailed understanding of factors controlling *FLM* gene expression and splicing will be obtained, it will be important to reexamine this aspect in more detail.

The role of *FLM* in flowering time variation was previously established in the temperature-insensitive accessions Nd-1 and Ei-6 where *FLM* is deleted [16, 17]. In our study, we report the first gene expression variation-allele for *FLM* and describe a molecular mechanism for the control of flowering time in ambient temperature through structural changes in *FLM* intron 1. It is interesting to note that loss-of-function alleles of flowering time genes are generally very rare and are typically distributed within a small geographic region. This may indicate that adaptation through gene loss may be disadvantageous outside of the specific ecological niche simply because the loss of the gene will prevent its future reactivation [59, 60]. In the case of *FLM*, this is exemplified by the Nd-1 and Ei-6 accessions, but also among the many known *FLC* alleles, only a few null alleles have been reported whereas gene expression-modulatory *FLC* alleles are more common [6, 12, 49, 50, 61]. In this regard, we perceive at least a trend towards a similar distribution among the *A*. *thaliana FLM* alleles. A deeper analysis about the *FLM* coding sequence polymorphisms will be required to make conclusive statements about the importance of strong and weak *FLM* alleles during adaptation of flowering time.

We conclude that structural variations of *FLM* intron 1 as described here represent an adaptive mechanism for the control of flowering time in *A*. *thaliana* and possibly also in other closely related *Brassicaceae*. *FLM* might have a very specific role in flowering time regulation because modulations of its expression seemingly affect only flowering and not other plant growth traits. We, therefore, think that *FLM* is an excellent candidate gene to precisely and steadily modulate flowering time in a dynamic manner over a broad range of temperature conditions to overcome the impacts of climate change on flowering in plants.

## Material and Methods

### Biological material

The following *A*. *thaliana* accessions and genotypes were used in this study and, unless stated otherwise, provided by the Nottingham Arabidopsis Stock Centre (NASC; Nottingham, UK): The Arabidopsis accessions Killean-0 (Kil-0), Columbia-0 (Col-0), and Nossen (No-0); the insertion mutants *flm-3* (Salk_141971; Col-0), GABI-KAT GK487H01 (GABI; Col-0), Salk_068360 (Salk; Col-0) as well as RIKEN-13-4593-1 (RIKEN; No-0) from the RIKEN Stock Center. In each case, the positions of the insertions were verified by DNA sequencing. The transgenic line gFLM^Col-0^ (*flm-3*) was previously described [19], *flc-3* and the line *FRI* ^*SF-2*^ *FLC* [6] were a gift from Franziska Turck and George Coupland (Max-Planck Institute of Plant Breeding Research, Cologne, Germany). A list of the *A*. *thaliana* accessions screened by PCR with the primers LP1, LP2, and RP1 for the *FLM* ^*LINE*^ structural polymorphism is provided as S6 Table. Primer sequences are provided as part of S7 Table.

### Physiological experiments

For flowering time analyses, plants were randomly arranged in trays and grown under constant white light (70–90 μmol m^-2^ s^-1^) or in long day-conditions with 16 hrs white light (110–130 μmol m^-2^ s^-1^)/8 hrs dark in MobyLux GroBanks (CLF Plant Climatics, Wertingen, Germany) or MLR-351 SANYO growth chambers (Ewald, Bad Nenndorf, Germany). Trays were rearranged every two days and water was supplied by subirrigation. Analysis of large plant sets was performed in a walk-in chamber with constant white light as described above. Flowering time was quantified by determining the time until the macroscopic appearance of the first flower bud (days to bolting, DTB) or by counting rosette and cauline leaf numbers (RLN, CLN). Student’s t-tests, ANOVA, and Tukey HSD tests were calculated with Excel (Microsoft) and R (http://www.r-project.org/), respectively.

### Kil-0 genome sequencing

For the resequencing of the Kil-0 genome or the late and early flowering F_3_ recombinant pools, libraries were prepared from 600 ng genomic DNA following the standard protocol of the TruSeq DNA Sample Preparation Kit v2 (Illumina, San Diego, CA). Paired-end sequencing with a read length of 100 bp was performed on a HiSeq 2500 (Illumina, San Diego, CA). Post-sequencing quality trimming was performed with the CLC Genomics Workbench (v. 7.0) and the following parameters: low quality limit = 0.05; ambiguous nucleotide = maximum 1; length minimum = 15. Post-trimming, 15 x 10^6^ and 20 x 10^6^ reads were obtained for the early and late flowering samples, respectively. Read mapping was performed using the TAIR10 release of the *A*. *thaliana* reference (The Arabidopsis Genome Initiative, 2000) genome reference sequence with the stringent settings: mismatch cost = 2; insertion cost = 2; deletion cost = 2; length fraction = 0.9; similarity = 0.9. An average 57- or 79-fold coverage was obtained from the early and late flowering DNA pools. Variant calling was performed using the probabilistic variant calling tool of the CLC Genomics Workbench (v. 7.0) and default settings. SNPs with a 30–120-fold coverage and frequency *f* > 20% as well as a presence call in both pooled samples were selected for allele frequency mapping. From those, SNPs that showed a frequency of < 80% in the resequencing analysis of the homozygous Kil-0 parental line were discarded. Smoothing using locally weighted scatterplot smoothing (LOESS) of SNP frequency values was achieved with R (http://www.r-project.org/). 95% confidence intervals, Δ*f* > 25%, and Δ*f*_*max*_ were calculated from the LOESS values. The *de novo* assembly of Kil-0 resequencing reads was performed using the CLC Genomics Workbench v. 7.0 with default settings. Contigs were identified by a simple search and were reassembled to the Col-0 genomic *FLM* sequence. The Kil-0 genomic sequence is available as LN866842 at www.ebi.ac.uk/ena.

### Mapping and backcrossing

To identify the causative locus for early flowering in Kil-0, the FT15 locus was mapped with polymorphic markers selected from a previously described marker collection [62]. Additional SSLP (single sequence length polymorphisms) markers were generated by searching the publicly available Kil-0 genomic sequence (www.1001genomes.org) or the genome sequence that was determined as part of this project for InDel (insertion/deletion) polymorphisms. PCR primers spanning these sites were designed with Primer3 [63] and tested on Col-0 and Kil-0 genomic DNA. The PCR fragments were generally between 200 and 700 bp long and separated on 2–3.5% agarose gels or using the QIAxcel Advanced Capillary Electrophoresis high resolution kit (Qiagen, Hilden, Germany). For rough mapping of FT15, ten early and ten late flowering F_2_ plants were selected from the extreme phenotypic borders of an F_2_ population (n = 124). The genetic marker distances were calculated from the genotype data of all screened F_2_ plants with JoinMap v.4.1 (Kyazma B.V.). A list of markers and the respective primers is provided as S8 Table.

Col^*FLM-Kil*^ and Kil^*flm-3*^ were generated by marker-assisted backcrosses. In brief, heterozygous F_1_ plants were genotyped with the primers LP1, LP2, and RP1 to examine the lines for the presence of the Col-0 or Kil-0 *FLM* allele and with Salk_141971 forward and reverse primers to test for the *flm-3* T-DNA insertion, respectively. Primer sequences are provided as part of S7 Table.

### Quantitative real-time PCR

For qRT-PCR analyses, total RNA was isolated from three biological replicates using the NucleoSpin RNA Plant kit (Machery-Nagel, Düren, Germany). DNA was removed by an on-column treatment with rDNase (Machery-Nagel, Düren, Germany). 2–3 μg total RNA were reverse transcribed with an oligo(dT) primer and M-MuLV Reverse Transcriptase (Fermentas, St. Leon-Rot, Germany) and the cDNA equivalent of 30–50 ng total RNA was used in a 10 μl PCR reaction with SsoAdvanced™ Universal SYBR Green Supermix (BioRad, München, Germany) in a CFX96 Real-Time System Cycler (BioRad, München, Germany). The relative quantification was calculated with the ΔΔCt method with *ACT8* as a control [64]. See S7 Table for a list of qRT-PCR primers.

### RNA-sequencing

To investigate significant gene expression differences between Col-0 and Kil-0, RNA was prepared from three biological replicate samples from ten day-old seedlings (21°C, long days) as described above. Sequencing libraries were prepared following the standard protocol of the TruSeq RNA Sample prep v2 Kit (Illumina, San Diego, CA). Paired-end sequencing with a read length of 100 bp was performed on a HiSeq 1000 (Illumina, San Diego, CA). RNA-seq reads from each biological replicate were then aligned against the Col-0 TAIR10 release genome using Bowtie (version 2.1.0) and Tophat2 (version 2.0.8) with default parameters [65, 66]. On the basis of the structural gene annotation for *A*. *thaliana* TAIR10, exon- and gene-level transcription was quantified by using HTSeq, differential expression tests were performed by using DESeq2 [67, 68]. Significantly differentially expressed genes were defined as genes with Benjamini-Hochberg-adjusted p-values < 0.01. Tests for differential exon usage were conducted on basis of RNA-seq exon counts with DEXseq [69]. The RNA-seq data are publically available as PRJEB9470 at www.ebi.ac.uk/ena.

A list of genes with a role in flowering time regulation was generated by searching the TAIR database (www.arabidopsis.org) for the term “flowering time”. The resulting list was reviewed manually and is presented in S3 Table. To subsequently analyze gene expression of the *FLM* locus at a nucleotide resolution, the corresponding Col-0 gene sequences were extracted from the Col-0 TAIR10 reference genome and the Kil-0 genome sequence as determined as part of this study. Subsequently, RNA-seq reads were aligned against the Kil-0 and Col-0 genomic sequences using Bowtie and Tophat2 as described above. The number of mapped RNA-seq reads from Col-0 and Kil-0 per nucleotide were counted with the toolset Bedtools and subsequently normalized to range between 0 (no expression) and 1 (maximum expression). For visualization, the mean expression level and 5% and 95% confidence intervals were determined across the biological replicates of one sample.

### Cloning procedures

To obtain a genomic *FLM* fragment from Kil-0 with a deletion of the 5.7 kb LINE insertion, a 6,981 bp g*FLM*(Kil-0)ΔLINE deletion fragment was amplified by overlap extension PCR from Kil-0 genomic DNA. Fragment 1 (bp—2367 to bp + 631) was amplified with ULC-1 and ULC-2 and fragment 2 (bp + 632 to bp + 4156 and 251 bp 3’-UTR plus 207 bp downstream sequence) with ULC-3 and ULC-4. After purification of the two subfragments, the full fragment was generated by overlap-extension PCR reaction with ULC-1 and ULC-4. The insert of the full-length fragment in pCR2.1-TOPO (Life Technologies, Carlsbad, CA) was sequenced and subcloned as a *Bam*HI and *Xho*I fragment into pGreen0229 [70].

To insert the LINE insertion into pDP34, a previously described construct with the Col-0 genomic *FLM* fragment p*FLM*::g*FLM* [19], pDP34 was mutagenized by PCR with ULC-12 to replace the sequence ATTGTTCA (bp +632 to bp +640) with a unique *Asc*I restriction site (GGCGCGCC) [71]. The LINE insertion was then amplified from Kil-0 genomic DNA with ULC-16 and ULC-17 and inserted as an *Asc*I fragment into the modified pDP34. All constructs were transformed into *Agrobacterium tumefaciens* strain GV3101 containing pSOUP and subsequently using floral dip transformation into Col-0, Kil-0, and *flm-3* [70, 72]. pDP79 and pDP80 were previously described [19]. T_1_ transformants were selected by spraying soil-grown plants with 0.1% BASTA. The list of primers is provided in S7 Table.

### *FLM* locus analysis of *A*. *thaliana* accessions

To test for the presence of the *FLM* ^*Kil-0*^ allele in a large collection of accessions, we performed PCR with the primers LP1, LP2, and RP1 on genomic DNA using Phusion (New England Biolabs, Frankfurt, Germany) or TaKaRa LA Taq polymerases (Takara Bio, Saint-Germain-en-Laye, France). Selected PCR products were analyzed by DNA sequencing following gel extraction. Sequencing primers are listed in S7 Table. A complete list of all accessions examined is provided as S6 Table.

Kil-0 genome sequence information was used to identify accessions with a LINE insertion by analyzing genome sequences as determined in the frame of the 1001 Arabidopsis genome sequencing project (www.1001genomes.org). To render read mapping specific for the Kil-0 allele, the insertion at the experimentally retrieved insertion sites flanked by 140 bp sequence was extracted for each of the two insertion breakpoints and defined as target sequences for mapping. Subsequently, all reads of the individual *A*. *thaliana* accessions were mapped against the target sequences using SHORE and genomemapper allowing for up to 5% single base pair differences including gaps with regard to the read length [73, 74]. To ensure that each supporting read spans at least 25% of its sequence across the insertion breakpoint, only reads that overlapped an insertion site with the inner 50% of its read sequence were counted as supporting an insertion breakpoint (core-mapping read). Furthermore, only unique mapped reads were included. An insertion was defined as present if there were at least two unique core-mapping reads at the left and right insertion breakpoint.

### LINE insert and *FLM* locus characterization

Several database searches with the Kil-0 LINE insert yielded no highly similar sequences, except for a 559 bp match to the second exon of Col-0 AT1G04625, which is most likely a transposon-related gene based on its ribonuclease H-like description. The LINE sequence within the Kil-0 insert was identified with RepeatMasker (http://www.repeatmasker.org) against PGSP-REdat, v_9.3_Eudicot (http://pgsb.helmholtz-muenchen.de/plant/recat/). The Pfam domains where annotated with hmmsearch of hmmer3 in all 6 reading frames against PfamA v27 [75, 76].

For phylogenetic analyses of the *FLM* locus, sequences of 88 randomly selected accessions were extracted from the *A*. *thaliana* 1001 genome project GEBowser (http://signal.salk.edu/atg1001/3.0/gebrowser.php) and aligned with sequences of the ten *FLM* ^*LINE*^ accessions and Col-0. Alignments were calculated using ClustalW and Neighbor-Joining trees (Maximum Composite Likelihood method, 1000 bootstrap replicates) were constructed with MEGA5 [77].

### 3’ RACE PCR

Polyadenylated transcripts were amplified according to [78]. Transcript pools were analyzed on an agarose gel and the upper and lower bands as depicted in S6E Fig were purified from all samples, subcloned into pCR2.1-TOPO (Life Technologies, Carlsbad, CA) and sequenced. Primers sequences are listed in S7 Table.

### Determination of *FLM* pre-mRNA abundance

Nuclei were isolated as previously described [79] with two biological replicates per line. RNA from nuclei pellet was extracted as previously described [80] and DNA was digested using DNaseI (Life Technologies, Carlsbad, CA). cDNA synthesis and qRT-PCR were performed as described above. Primer sequences are provided in S7 Table.

## Supporting Information

S1 Fig Flowering time analysis in Kil-0 and Col-0. **(A)** and **(B)** Quantitative flowering time analysis of Col-0 and Kil-0 in different growth conditions. LD, long days; SD, short days; CC, continuous light. **(C)** Vernalization response of Col-0 and Kil-0. Total leaf number of vernalized plants (8 weeks at 4°C) compared to non-vernalized plants. **(D)** qRT-PCR analysis of *FLC* of 12 day-old non-vernalized plants grown in 21°C and long day photoperiod. Kas-1 was included as a vernalization-sensitive accession.
(TIF)

S2 Fig Characterization of the Kil-0 x Col-0 mapping population. **(A)** Representative photographs of Kil-0 x Col-0 F_2_ plants grown at 15°C and selected from the extreme phenotypic borders. **(B)** Rough mapping analysis using ten early and ten late flowering F_2_ plants as shown in (A). The average Kil-0 allele frequency of the early or late flowering plants is shown for twenty SSLP markers. Markers were selected from [62] or generated as described. Significance of the two associations is indicated with * = p ≤ 0.05; ** p ≤ 0.01. **(C)** Schematic representation of the FT15 mapping procedure. 43 recombinant plants with the genotypes M1^Kil-0/Col-0^/M2^Kil-0/Kil-0^ or M1^Kil-0/Kil-0^/M2^Kil-0/Col-0^ in the 968 kb interval were selected from 1049 F_2_ plants. After selfing, 43 F_3_ recombinant lines were obtained and four plants per F_3_ family were individually genotyped and phenotyped at 15°C with additional markers identifying a 151 kb peak region. Bulks of early or late flowering F_3_ plants with a recombination in this region were sequenced. **(D)** Quantitative flowering time measured in days to bolting of 127 F_3_ recombinant plants from 43 families grown in 15°C and continuous light. Each bar represents one plant. Averages ± SD of 15 Col-0 and Kil-0 plants are indicated as blue and red bars, respectively. **(E)** Results of the mapping procedure described in (A). The 968 kb interval with flanking marker M1 and M2 and seven internal markers is shown as a vertical line with the genetic [cM] and physical [kb] marker distances as calculated based on the 1049 F_2_ individuals and the chromosomal position. The middle panel shows the respective allele frequency of the selected 309 SNPs within either the early or the late flowering plant pool. Red and blue lines show the respective LOESS-smoothed allele frequency values with the 95%-confidence interval shown as a grey dotted line. Vertical black lines represent the final mapping interval of 31.3 kb with Δ*f* > 25%. The SNP with the highest difference between early and late pool (Δ*f*_max_) is marked with a black dotted line. The lower panel shows a detailed illustration of the eleven annotated gene models in the 31.3 kb interval. **(F)** Physical distribution of the 309 SNPs along the 151 kb mapping interval.
(TIF)

S3 Fig Detailed description of the inserted sequence in intron 1 of *FLM* ^*Kil-0*^. The light grey bar indicates the complete 5.7 kb insertion. The dark blue bar indicates the partition of the insertion that shows 68% identity to the AT_LINE1-8 retrotransposon sequence.
(TIF)

S4 Fig Flowering time analysis of *flm-3* in different ambient temperatures. Total leaf number [TLN] of *flm-3* plants (n = 20–30) was compared to Col-0 plants (n = 20–30).
(TIF)

S5 Fig Analysis of flowering time gene expression. **(A)** and **(B)** qRT-PCR analyses of *SVP* and *FT* transcript abundance at 21°C during a 24 h long day photoperiod. Fold changes are averages ± SE of three measurements. **(C)**, **(D)**, and **(E)** qRT-PCR analyses of *FT*, *SOC1*, and *SVP* transcript abundance at 21°C in long day photoperiod. Samples were taken at ZT16 from 6, 9, 13, 15 day-old plants. Fold changes are averages ± SE of three measurements.
(TIF)

S6 Fig Analysis of *FLM* processing (**A**) Images of agarose gels illustrating the absence of any spliced transcripts from the nuclear RNA preparations since the intron-spanning ACT8 primers only amplify a 254 bp fragment corresponding to the non-spliced fragment but not the 111 bp fragment corresponding to the spliced form. (**B**) The absence of genomic DNA contamination was determined by a primer located downstream from the FLM 3’ UTR. Genomic DNA samples with different DNA template concentrations are shown as positive controls. (**C**) Semi-quantitative PCR with primers amplifying the full-length FLM gene using two cDNA samples each from ten days-old Col-0 and Kil-0 plants grown at 21°C. (**D**) qRT-PCR analyses of intron 1 sequence-containing transcripts, which were amplified using the reverse primers 1–6 as indicated in (Fig 4I). Fold changes are averages + SE of three biological replicates. Note that the expression value comparisons between the fragments are approximations since the primer efficiencies are not exactly identical. (**E**) Image of an agarose gel with the analysis of 3’ RACE PCR products. The respective upper and lower fragments were isolated for sequencing. (**F**), and (**G**) qRT-PCR analyses of ten day-old seedlings grown under 21°C long-day conditions without treatment or following a 5 hr mock or 20 μM CHX treatment, respectively. Fragment 3 and 4 correspond to fragment 3 and 4 depicted in Fig 4I. Fold changes are averages ± SE of three biological replicates. Student’s t-tests were performed as indicated: * = p ≤ 0.05; ** ≤ 0.01. All primer sequences are provided in S7 Table.
(TIF)

S7 Fig Geographic distribution of the accessions used (A) for the PCR-based screen and (B) for the analysis of unmapped reads. (TIF)

S8 Fig Neighbour-Joining tree of *FLM* ^*LINE*^ accessions and randomly selected accessions. (TIF)

S9 Fig Phenotypic and molecular analysis of *FLM* ^*LINE*^ accessions. **(A)** and **(B)** Representative photographs of the *FLM* ^*LINE*^ accessions grown at 21°C and 15°C in long day photoperiod. **(C)** Alignment of the *FLM* locus and 1.8 kb upstream sequence of the *FLM* ^*LINE*^ accessions. Polymorphic sites between the *FLM* ^*LINE*^ accessions are indicated with an asterisk. The 5.7 kb insertion of *FLM* ^*Kil-0*^ at position +631 is not represented here. **(D)** Multiple alignment of the 5.7 kb inserted sequence of the *FLM* ^LINE^ accessions. Black vertical lines indicate SNPs, light grey horizontal lines indicate insertions and deletions.
(TIF)

S10 Fig *FLM* introns are essential for *FLM* expression. **(A)** Schematic representation of the constructs used for the analysis. **(B)** Averages ± SD from two biological replicates of qRT-PCR analyses of *FLM-ß and FLM-δ* from four independent T_2_ lines transformed with the respective constructs. **(C)** Quantitative flowering time analysis of independent T_1_ transformants with the constructs described in (A).
(TIF)

S11 Fig *FLM* alleles do not control plant height. Final plant height of plants grown at 21°C and 15°C under long day conditions.
(TIF)

S1 Table List of plants with an early and late flowering phenotype that were pooled for next generation sequencing. (XLS)

S2 Table List of genes that are annotated in the 31.3 kb mapping interval. (XLS)

S3 Table List of genes with an implied role in flowering time regulation and analysis of their differential regulation by RNA-seq. (XLS)

S4 Table Annotation of the LINE insertion identified in the first intron of *FLM* from Kil-0. (XLS)

S5 Table List of accessions carrying the *FLM* ^*Kil-0*^ allele. (XLS)

S6 Table List of accessions screened for the Kil-0 insertion. (XLS)

S7 Table List of primers used for cloning, genotyping, qRT, and sequencing. (XLS)

S8 Table List of primers for marker detection. (XLS)
