# Supplementary figures and images for: Modulation of Ambient Temperature-Dependent Flowering in Arabidopsis thaliana by Natural Variation of FLOWERING LOCUS M
Source: PLoS Genet. 2015 Oct 22;11(10):e1005588. doi: 10.1371/journal.pgen.1005588 (PMC4619661; doi:10.1371/journal.pgen.1005588)

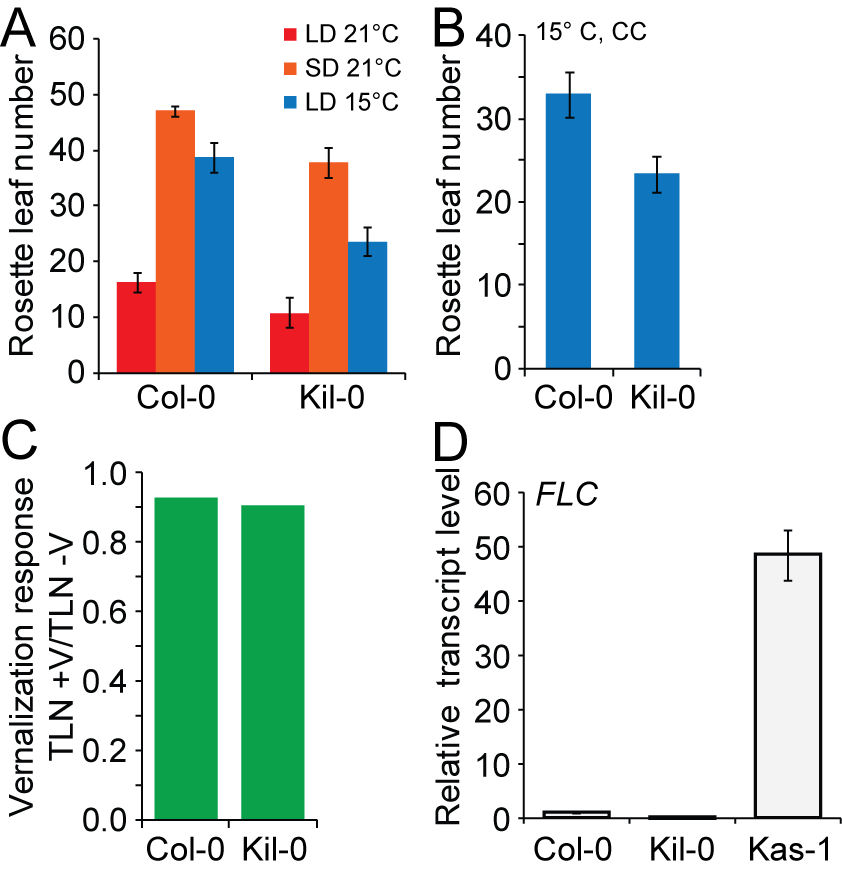

Supplement: S1 Fig — (A) and (B) Quantitative flowering time analysis of Col-0 and Kil-0 in different growth conditions. LD, long days; SD, short days; CC, continuous light. (C) Vernalization response of Col-0 and Kil-0. Total leaf number of vernalized plants (8 weeks at 4°C) compared to non-vernalized plants. (D) qRT-PCR analysis of FLC of 12 day-old non-vernalized plants grown in 21°C and long day photoperiod. Kas-1 was included as a vernalization-sensitive accession. (TIF) [file pgen.1005588.s001.tif]

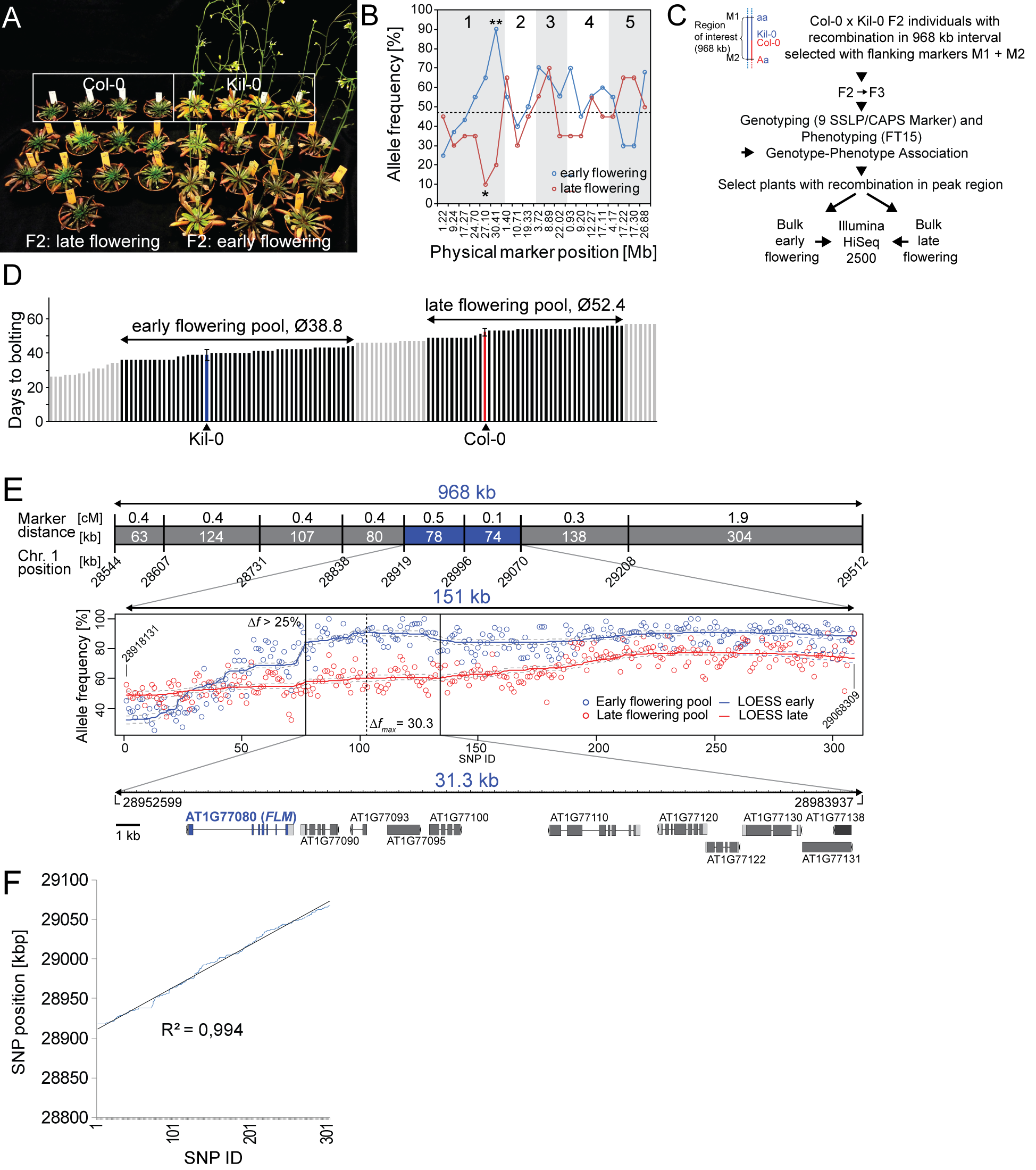

Supplement: S2 Fig — (A) Representative photographs of Kil-0 x Col-0 F2 plants grown at 15°C and selected from the extreme phenotypic borders. (B) Rough mapping analysis using ten early and ten late flowering F2 plants as shown in (A). The average Kil-0 allele frequency of the early or late flowering plants is shown for twenty SSLP markers. Markers were selected from [62] or generated as described. Significance of the two associations is indicated with * = p ≤ 0.05; ** p ≤ 0.01. (C) Schematic representation of the FT15 mapping procedure. 43 recombinant plants with the genotypes M1Kil-0/Col-0/M2Kil-0/Kil-0 or M1Kil-0/Kil-0/M2Kil-0/Col-0 in the 968 kb interval were selected from 1049 F2 plants. After selfing, 43 F3 recombinant lines were obtained and four plants per F3 family were individually genotyped and phenotyped at 15°C with additional markers identifying a 151 kb peak region. Bulks of early or late flowering F3 plants with a recombination in this region were sequenced. (D) Quantitative flowering time measured in days to bolting of 127 F3 recombinant plants from 43 families grown in 15°C and continuous light. Each bar represents one plant. Averages ± SD of 15 Col-0 and Kil-0 plants are indicated as blue and red bars, respectively. (E) Results of the mapping procedure described in (A). The 968 kb interval with flanking marker M1 and M2 and seven internal markers is shown as a vertical line with the genetic [cM] and physical [kb] marker distances as calculated based on the 1049 F2 individuals and the chromosomal position. The middle panel shows the respective allele frequency of the selected 309 SNPs within either the early or the late flowering plant pool. Red and blue lines show the respective LOESS-smoothed allele frequency values with the 95%-confidence interval shown as a grey dotted line. Vertical black lines represent the final mapping interval of 31.3 kb with Δf > 25%. The SNP with the highest difference between early and late pool (Δf max) is marked with a black dotted line. [file pgen.1005588.s002.tif]

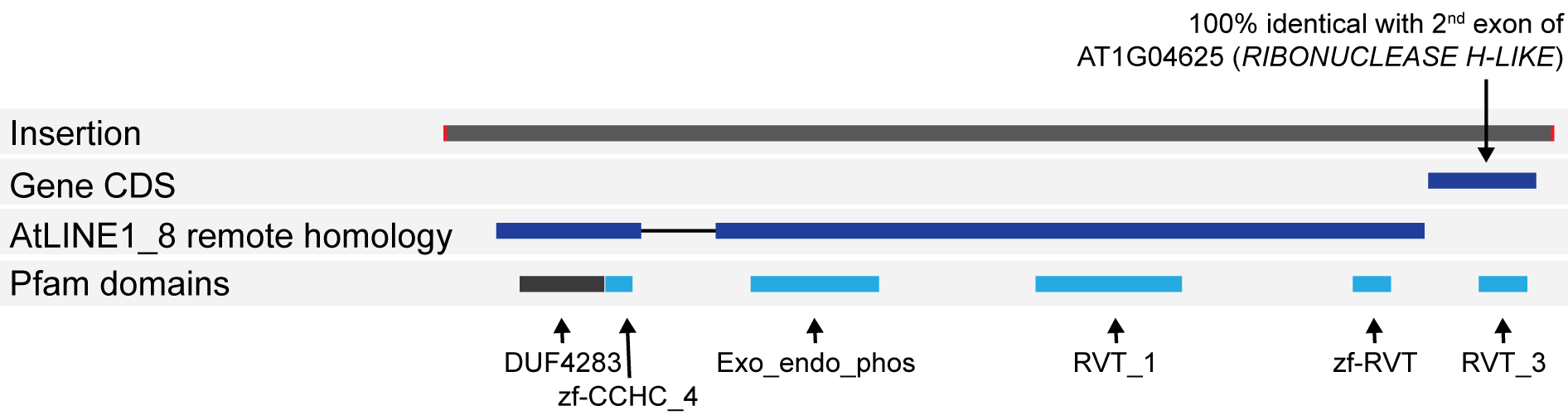

Supplement: S3 Fig — The light grey bar indicates the complete 5.7 kb insertion. The dark blue bar indicates the partition of the insertion that shows 68% identity to the AT_LINE1-8 retrotransposon sequence. (TIF) [file pgen.1005588.s003.tif]

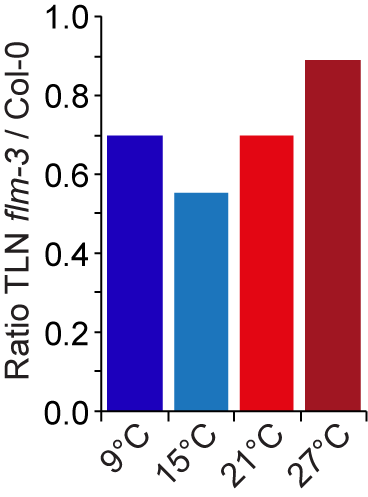

Supplement: S4 Fig — Total leaf number [TLN] of flm-3 plants (n = 20–30) was compared to Col-0 plants (n = 20–30). (TIF) [file pgen.1005588.s004.tif]

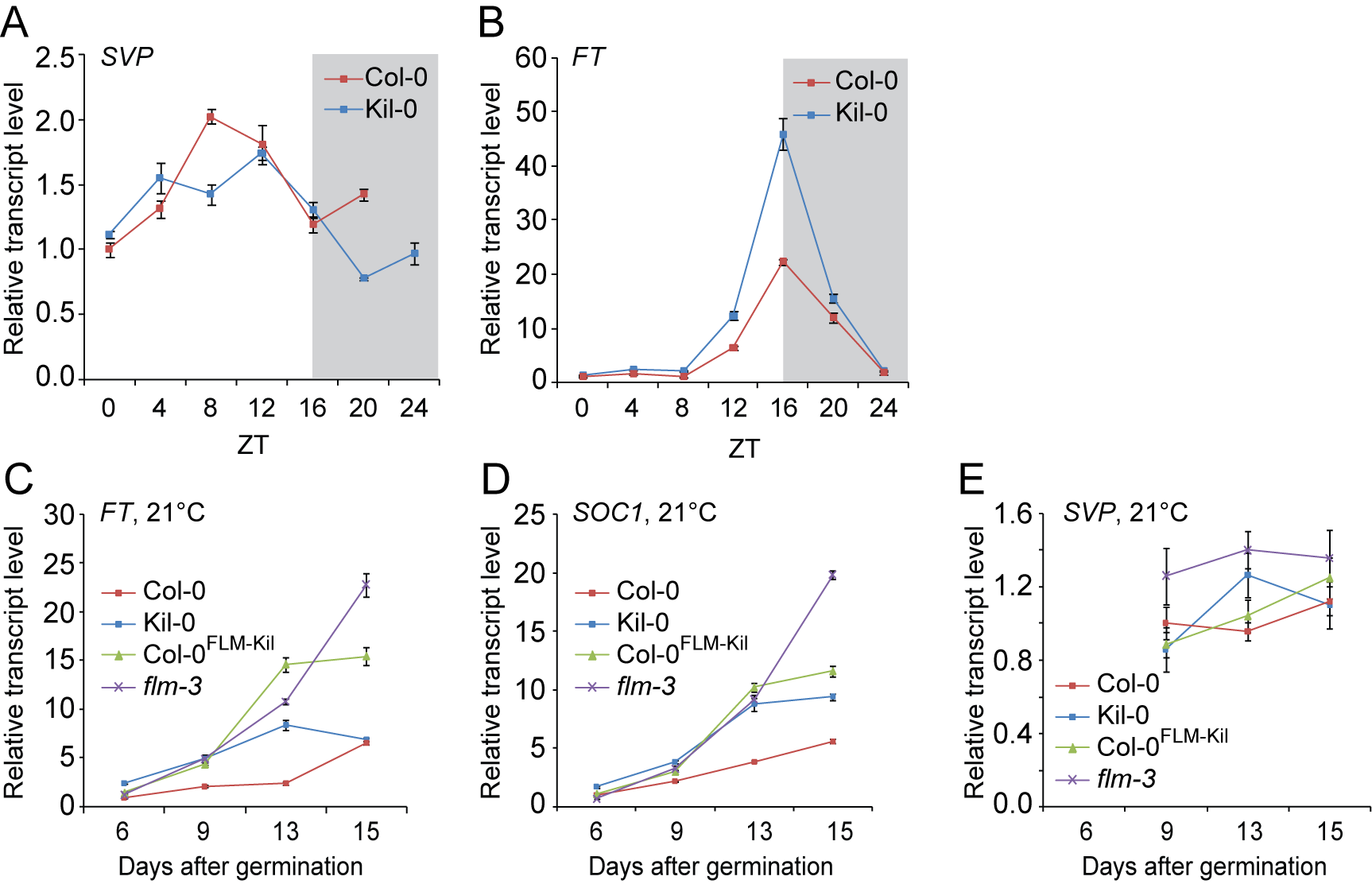

Supplement: S5 Fig — (A) and (B) qRT-PCR analyses of SVP and FT transcript abundance at 21°C during a 24 h long day photoperiod. Fold changes are averages ± SE of three measurements. (C), (D), and (E) qRT-PCR analyses of FT, SOC1, and SVP transcript abundance at 21°C in long day photoperiod. Samples were taken at ZT16 from 6, 9, 13, 15 day-old plants. Fold changes are averages ± SE of three measurements. (TIF) [file pgen.1005588.s005.tif]

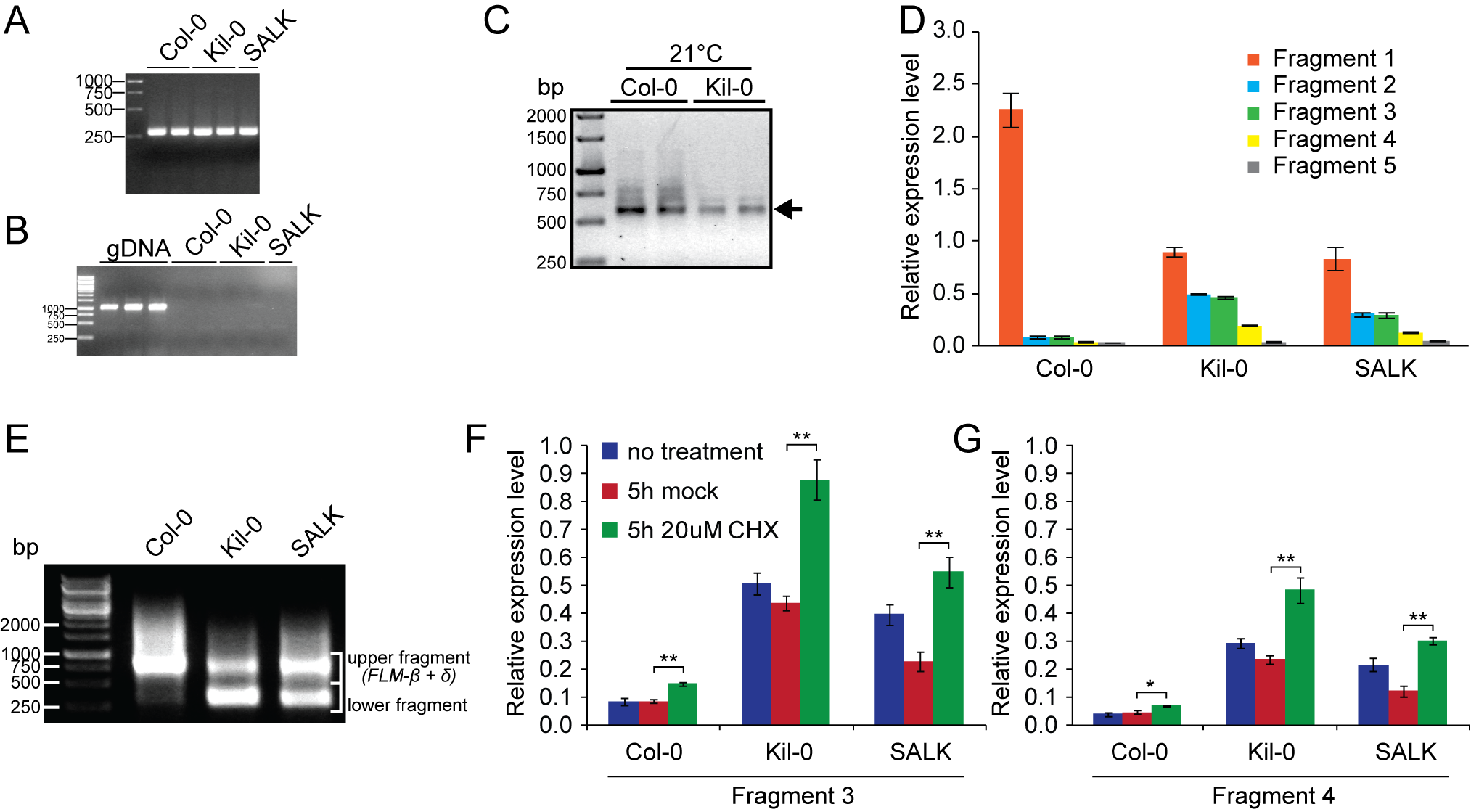

Supplement: S6 Fig — (A) Images of agarose gels illustrating the absence of any spliced transcripts from the nuclear RNA preparations since the intron-spanning ACT8 primers only amplify a 254 bp fragment corresponding to the non-spliced fragment but not the 111 bp fragment corresponding to the spliced form. (B) The absence of genomic DNA contamination was determined by a primer located downstream from the FLM 3’ UTR. Genomic DNA samples with different DNA template concentrations are shown as positive controls. (C) Semi-quantitative PCR with primers amplifying the full-length FLM gene using two cDNA samples each from ten days-old Col-0 and Kil-0 plants grown at 21°C. (D) qRT-PCR analyses of intron 1 sequence-containing transcripts, which were amplified using the reverse primers 1–6 as indicated in (Fig 4I). Fold changes are averages + SE of three biological replicates. Note that the expression value comparisons between the fragments are approximations since the primer efficiencies are not exactly identical. (E) Image of an agarose gel with the analysis of 3’ RACE PCR products. The respective upper and lower fragments were isolated for sequencing. (F), and (G) qRT-PCR analyses of ten day-old seedlings grown under 21°C long-day conditions without treatment or following a 5 hr mock or 20 μM CHX treatment, respectively. Fragment 3 and 4 correspond to fragment 3 and 4 depicted in Fig 4I. Fold changes are averages ± SE of three biological replicates. Student’s t-tests were performed as indicated: * = p ≤ 0.05; ** ≤ 0.01. All primer sequences are provided in S7 Table. (TIF) [file pgen.1005588.s006.tif]

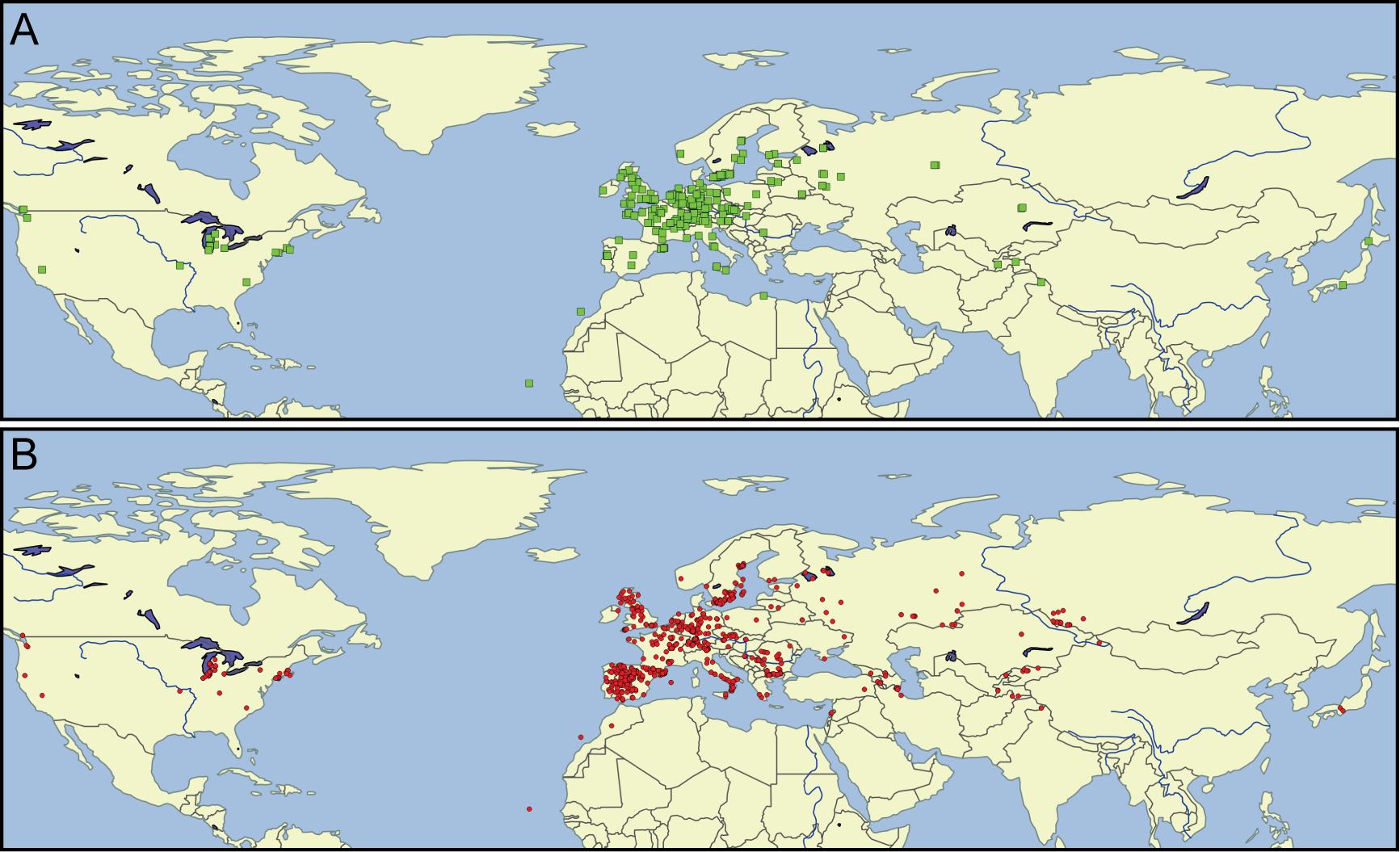

Supplement: S7 Fig — (TIF) [file pgen.1005588.s007.tif]

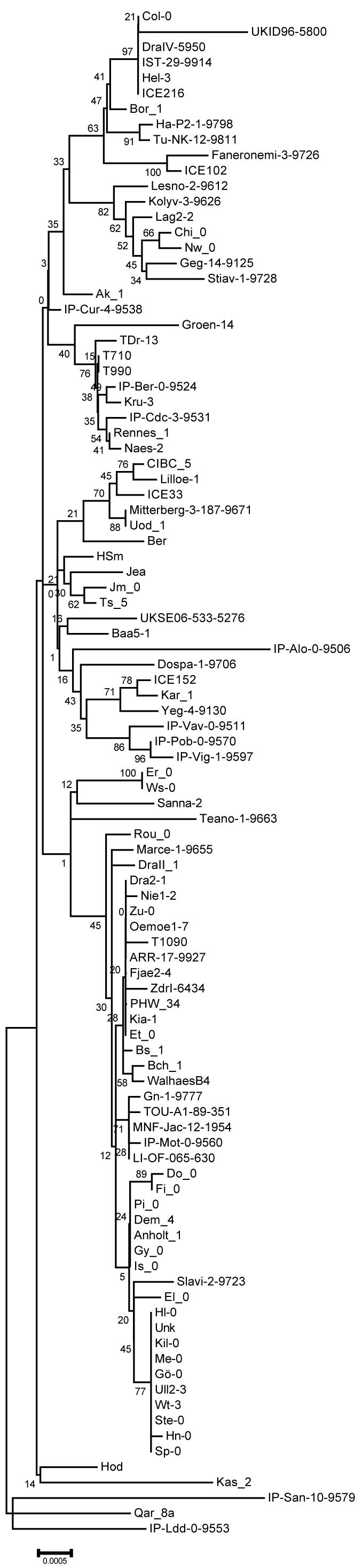

Supplement: S8 Fig — (TIF) [file pgen.1005588.s008.tif]

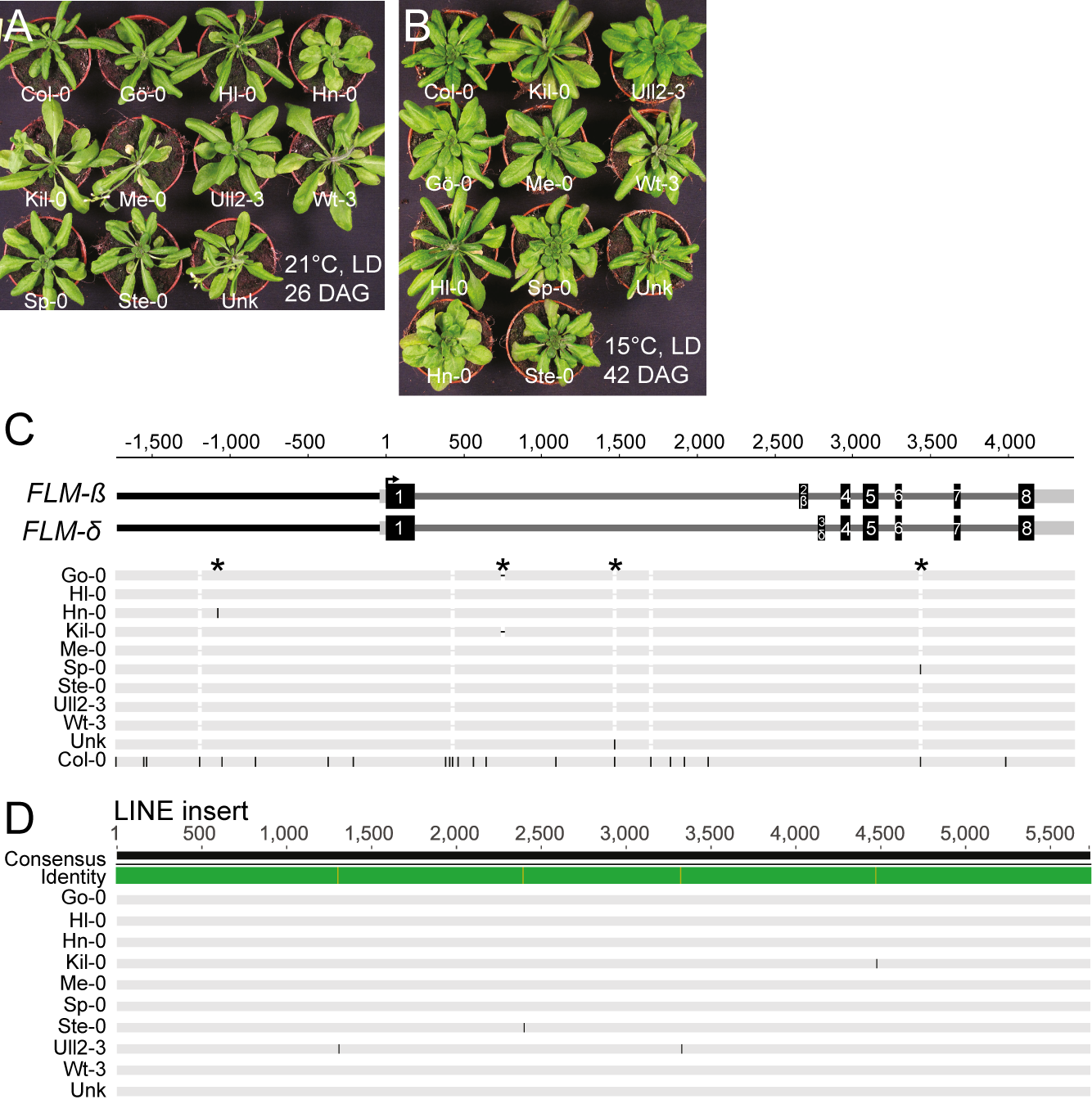

Supplement: S9 Fig — (A) and (B) Representative photographs of the FLM LINE accessions grown at 21°C and 15°C in long day photoperiod. (C) Alignment of the FLM locus and 1.8 kb upstream sequence of the FLM LINE accessions. Polymorphic sites between the FLM LINE accessions are indicated with an asterisk. The 5.7 kb insertion of FLM Kil-0 at position +631 is not represented here. (D) Multiple alignment of the 5.7 kb inserted sequence of the FLM LINE accessions. Black vertical lines indicate SNPs, light grey horizontal lines indicate insertions and deletions. (TIF) [file pgen.1005588.s009.tif]

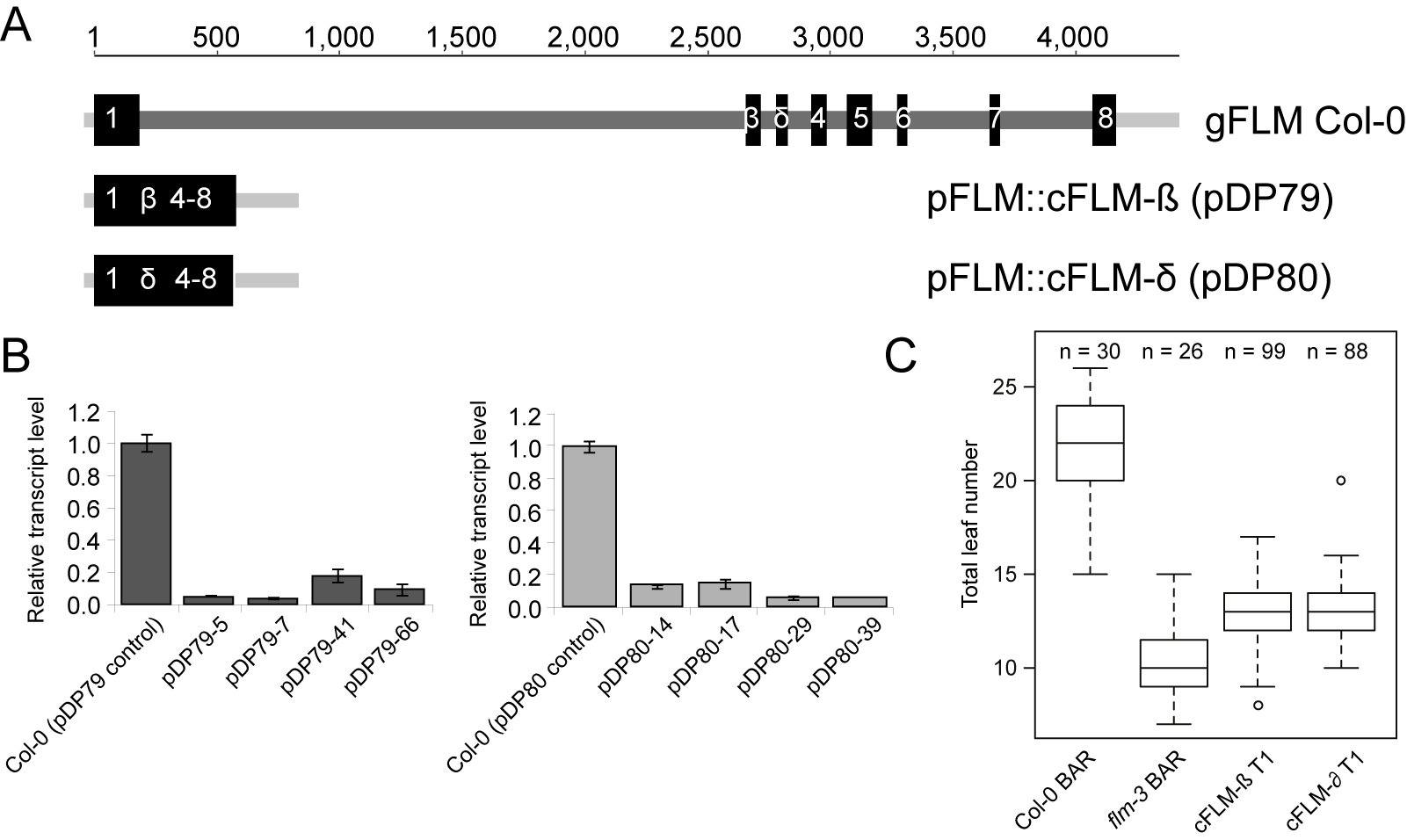

Supplement: S10 Fig — (A) Schematic representation of the constructs used for the analysis. (B) Averages ± SD from two biological replicates of qRT-PCR analyses of FLM-ß and FLM-δ from four independent T2 lines transformed with the respective constructs. (C) Quantitative flowering time analysis of independent T1 transformants with the constructs described in (A). (TIF) [file pgen.1005588.s010.tif]

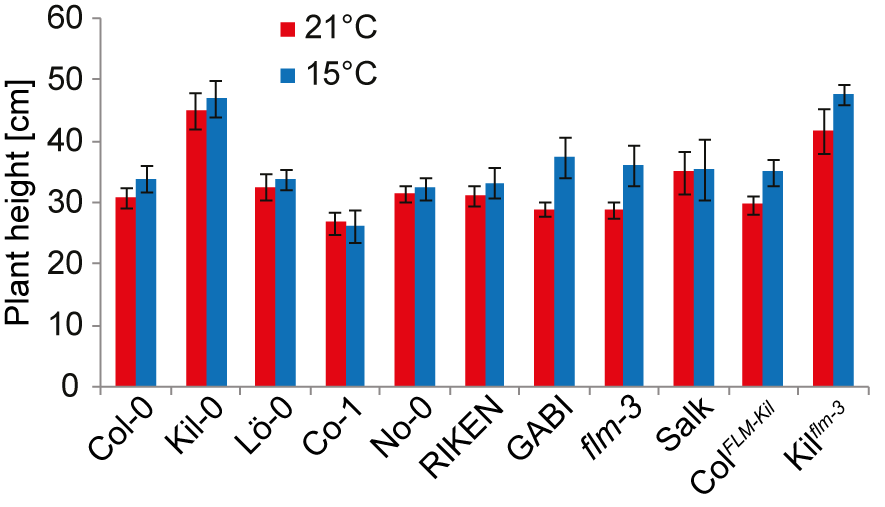

Supplement: S11 Fig — Final plant height of plants grown at 21°C and 15°C under long day conditions. (TIF) [file pgen.1005588.s011.tif]
